# Supplementary material for: YTHDF2 regulates self non-coding RNA metabolism to control inflammation and tumorigenesis
Source: Nat Commun. 2025 Nov 12;16:9946. doi: 10.1038/s41467-025-64898-7 (PMC12612255; doi:10.1038/s41467-025-64898-7)
Supplement: Supplementary file 1 — Supplementary Information [file 41467_2025_64898_MOESM1_ESM.pdf]

YTHDF2 regulates self non-coding RNA metabolism to control inflammation and tumorigenesis

Yang et al.

Supplementary figures, figure legends, and tables

Supplementary figures and figure legends

A The top 30 pathways in 764 Enriched Ontology Clusters

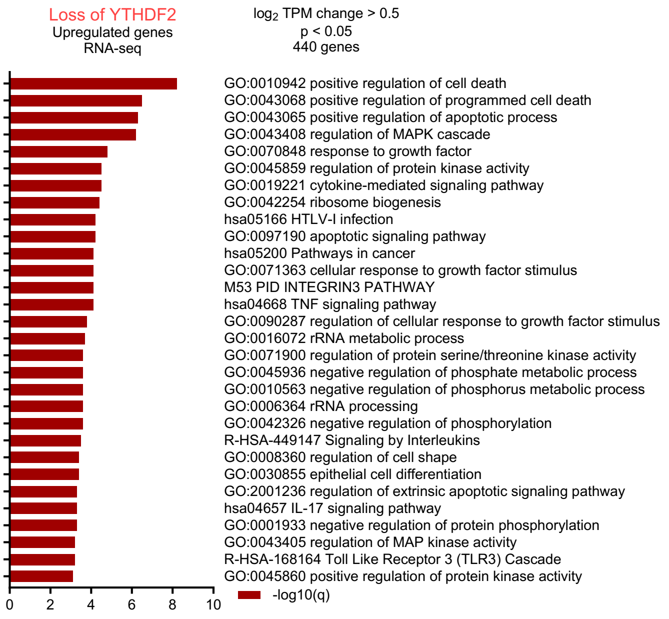

B

Rank	UVB Irradiation & RNA seq	
1	ncRNA metabolic process	GO:0034660
2	Metabolism of RNA	R-HSA-8953854
3	ribonucleoprotein complex biogenesis	GO:0022613
4	ribosome biogenesis	GO:0042254
5	ncRNA processing	GO:0034470
6	rRNA metabolic process	GO:0016072
7	rRNA processing	GO:0006364
8	mitochondrial gene expression	GO:0140053
9	Cell Cycle	R-HSA-1640170
10	mitochondrial translation	GO:0032543
11	tRNA metabolic process	GO:0006399
12	Cell Cycle, Mitotic	R-HSA-69278
13	Processing of Capped Intron-Containing Pre-mRNA	R-HSA-72203
14	Translation	R-HSA-72766
15	mRNA processing	GO:0006397
16	rRNA processing	R-HSA-72312
17	RNA splicing	GO:0008380
18	translational termination	GO:0006415
19	translation	GO:0006412
20	rRNA processing in the nucleus and cytosol	R-HSA-8868773
21	peptide biosynthetic process	GO:0043043
22	DNA replication	GO:0006260
23	Mitochondrial translation elongation	R-HSA-5389840
24	mRNA Splicing - Major Pathway	R-HSA-72163
25	Major pathway of rRNA processing in the nucleolus and cytosol	R-HSA-6791226
26	mitochondrial translational elongation	GO:0070125
27	Mitochondrial translation	R-HSA-5368287
28	mRNA Splicing	R-HSA-72172
29	Mitochondrial translation initiation	R-HSA-5368286
30	rRNA modification in the nucleus and cytosol	R-HSA-6790901

YTHDF2 IP & Quantative Interactome		Rank
R-HSA-8953854	Metabolism of RNA	1
R-HSA-8868773	rRNA processing in the nucleus and cytosol	2
R-HSA-72312	rRNA processing	3
R-HSA-6791226	Major pathway of rRNA processing in the nucleolus and cytosol	4
R-HSA-72766	Translation	5
GO:0006412	translation	6
GO:0043043	peptide biosynthetic process	7
CORUM:3055	Nop56p-associated pre-rRNA complex	8
GO:0043604	amide biosynthetic process	9
GO:0022613	ribonucleoprotein complex biogenesis	10
GO:0006518	peptide metabolic process	11
R-HSA-72706	GTP hydrolysis and joining of the 60S ribosomal subunit	12
hsa03010	Ribosome	13
GO:0002181	cytoplasmic translation	14
R-HSA-72737	Cap-dependent Translation Initiation	15
R-HSA-72613	Eukaryotic Translation Initiation	16
R-HSA-168255	Influenza Infection	17
R-HSA-156827	L13a-mediated translational silencing of Ceruloplasmin expression	18
R-HSA-1799339	SRP-dependent cotranslational protein targeting to membrane	19
R-HSA-975956	Nonsense Mediated Decay (NMD) independent of the Exon Junction Complex (EJC)	20
R-HSA-72689	Formation of a pool of free 40S subunits	21
R-HSA-975957	Nonsense Mediated Decay (NMD) enhanced by the Exon Junction Complex (EJC)	22
R-HSA-927802	Nonsense-Mediated Decay (NMD)	23
GO:0042254	ribosome biogenesis	24
R-HSA-168273	Influenza Viral RNA Transcription and Replication	25
R-HSA-72764	Eukaryotic Translation Termination	26
CORUM:306	Ribosome, cytoplasmic	27
R-HSA-192823	Viral mRNA Translation	28
R-HSA-156902	Peptide chain elongation	29
R-HSA-9633012	Response of EIF2AK4 (GCN2) to amino acid deficiency	30

Supplementary Figure S1. Related to Figure 1. Pathway analysis of UVB-induced genes and YTHDF2-interacting proteins using Metascape.

A. List of top 30 enriched pathways and 2 additional antiviral pathways from genes that are up-regulated by YTHDF2 knockdown. Genes with an adjusted *p* value less than 0.05 by DESeq2 were used for pathway analysis.

B. Shared pathways from the top 30 enriched pathways for 2471 genes up-regulated by UVB irradiation and the top 30 enriched pathways for YTHDF2-interacting 761 proteins, with red lines indicating the same pathways and blue lines indicating similar pathways. Genes with an adjusted *p* value less than 0.05 by DESeq2 were used for pathway analysis.

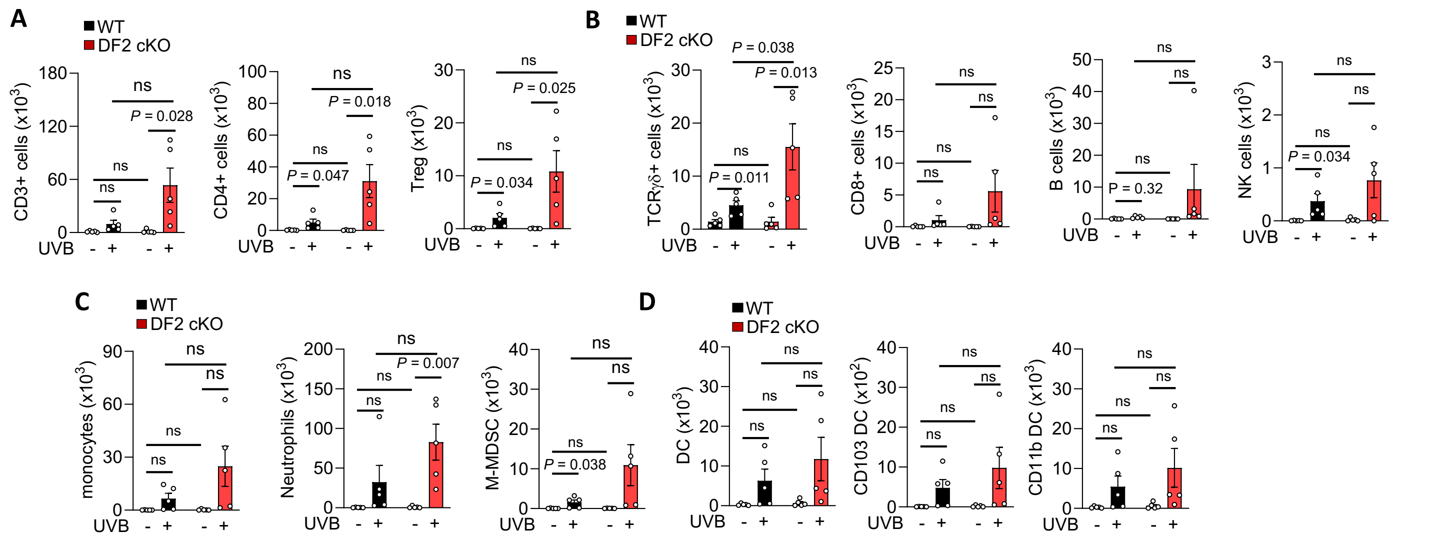

Supplementary Figure S2. Related to Figure 1. Skin YTHDF2 regulates immune cell abundance in the skin.

A-B. Number of CD3$^+$, CD4$^+$ T (CD3$^+$CD4$^+$), Tregs (CD3$^+$CD4$^+$FOXP3$^+$), TCR γδ$^+$ T (CD45$^+$CD3$^+$TCR γδ$^+$), CD8$^+$ T (CD3$^+$CD8$^+$), B (CD3$^-$B220$^+$), and NK cells (CD3$^-$NK1.1$^+$) in the skin of sham- and UVB-irradiated WT and DF2 cKO mice.

C. Number of monocytes (CD11b$^+$Ly6C$^+$Ly6G$^-$), neutrophils (CD11b$^+$Ly6C$^-$Ly6G$^+$), and M-MDSC (CD11b$^+$Ly6C$^+$Ly6G$^-$MHCII$^-$) cells in the skin as in A-C.

D. Number of DC (Ly6C$^-$MHCII$^+$CD11C$^+$), CD103$^+$ DC (Ly6C$^-$MHCII$^+$CD11C$^+$CD103$^+$CD11b$^-$), and CD11b$^+$ DC (Ly6C$^-$MHCII$^+$CD11C$^+$CD103$^-$CD11b$^+$) cells in the skin as in A-C.

Statistical analyses were performed using two-tailed unpaired Student's t-test. Mean ± SE are shown; n = 5 mice per group (A–D). ns, not significant. All experiments were conducted using biologically independent samples.

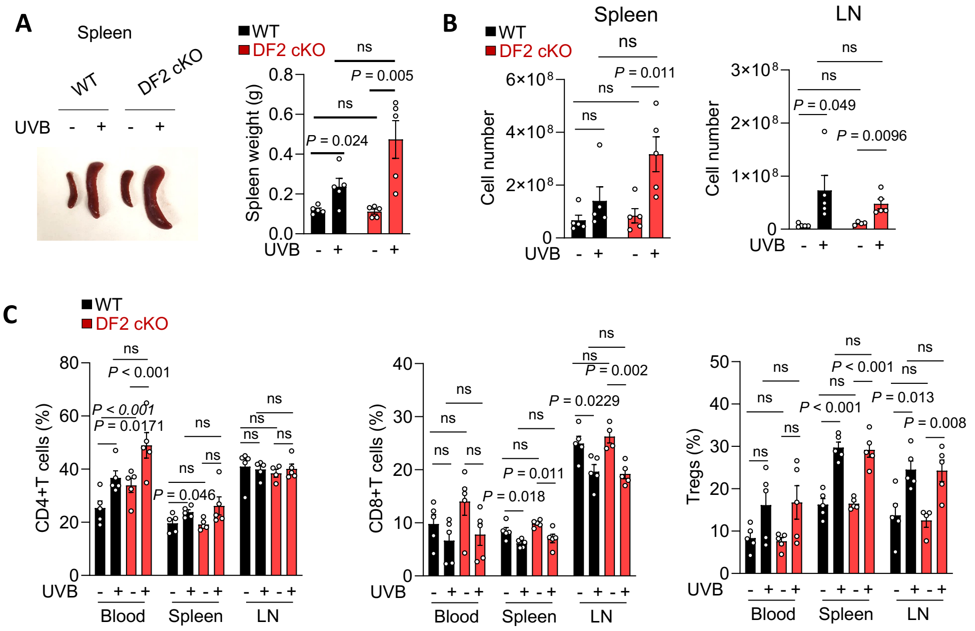

Supplementary Figure S3. Related to Figure 1. Skin YTHDF2 controls immune cell abundance in the spleen, peripheral blood, and lymph node (LN).

A. Representative images of spleen and quantification of spleen weight.
B. Quantitation of cell numbers in the spleen and LN.
C. Quantitation of $CD4^+$ T ($CD3^+CD4^+$), $CD8^+$ T ($CD3^+CD8^+$), Tregs ($CD3^+CD4^+FOXP3^+$) cells in the blood, spleen, and LN.
Statistical analyses were conducted using two-tailed unpaired Student's t-test . Mean ± SE are shown; n = 5 mice per group (A–C). ns, not significant (P > 0.05). All experiments were conducted using biologically independent samples.

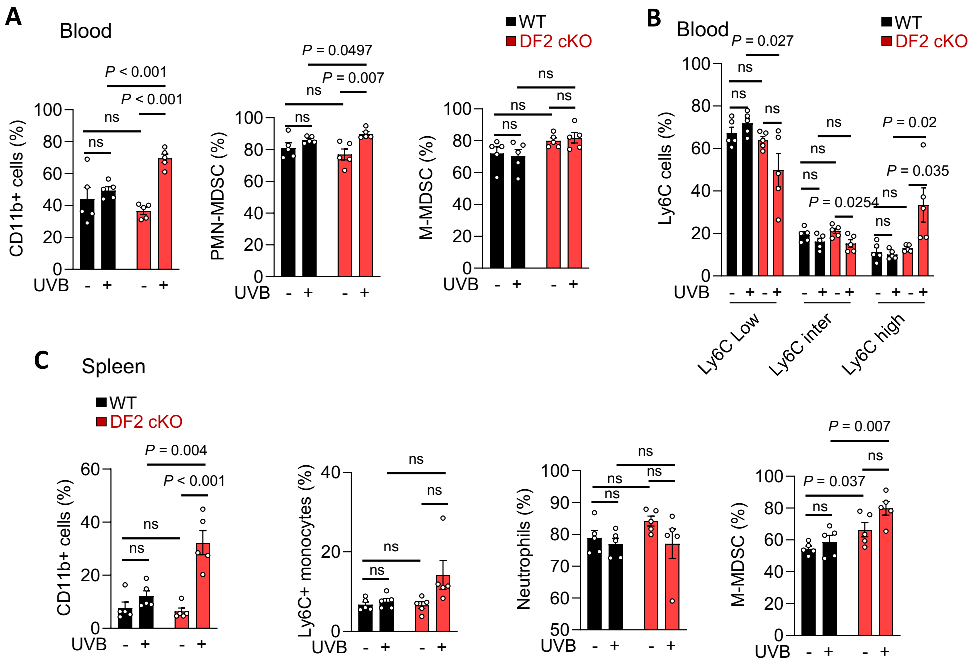

Supplementary Figure S4. Related to Figure 1. Skin YTHDF2 controls immune cell abundance in the spleen and peripheral blood.

A. Quantitation of CD11b$^+$ cells, PMN-MDSC, and M-MDSC in the blood.

B. Quantitation of CD11b$^+$ Ly6C cells in the blood.

C. Quantitation of CD11b$^+$ cells, Ly6C$^+$ monocytes, neutrophils, and M-MDSCs in the spleen.

Statistical analyses were conducted using two-tailed unpaired Student's t-test . Mean ± SE are shown; n = 5 mice per group (A–C). ns, not significant (P > 0.05). All experiments were conducted using biologically independent samples.

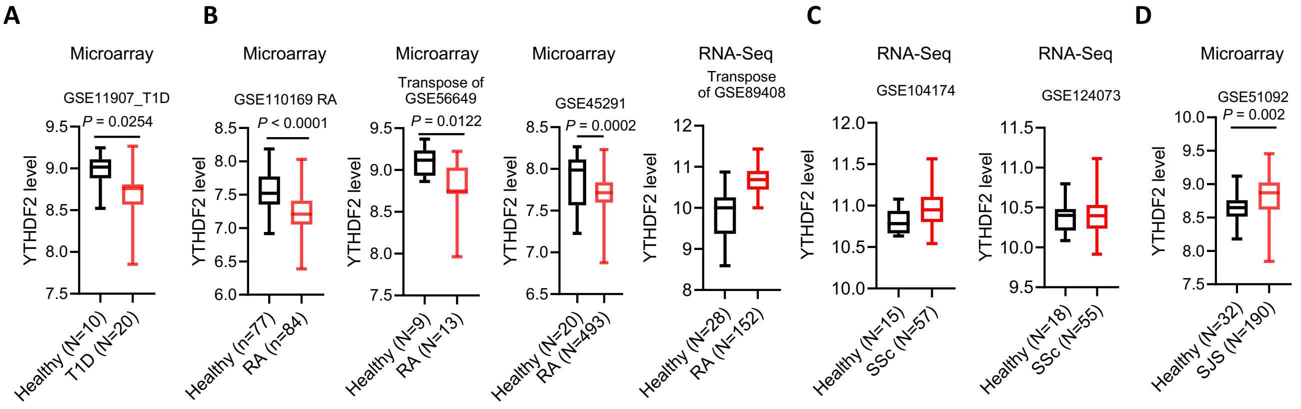

Supplementary Figure S5. Related to Figure 1. YTHDF2 expression in human autoimmune diseases.

A–D. Box plots of YTHDF2 expression in Type 1 diabetes (A), rheumatoid arthritis (B), systemic sclerosis (C), and Sjögren's syndrome (D). In the box plots, the centre line represents the median, the bounds of the box represent the interquartile range, and the whiskers extend to the minimum and maximum values.
Statistical analyses were conducted using two-tailed unpaired Student's t-test (for microarray analyses). ns, not significant (P > 0.05).

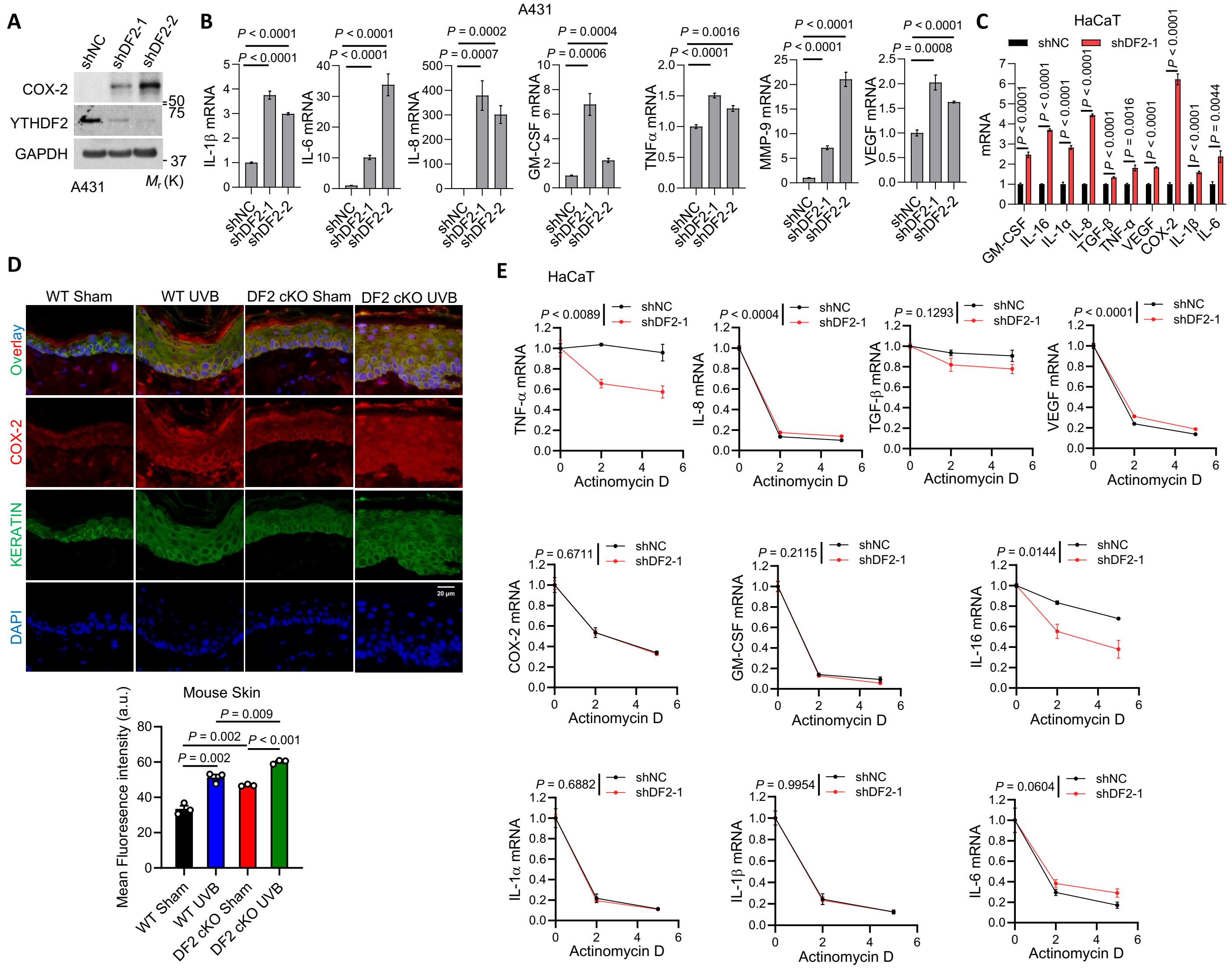

Supplementary Figure S6. Related to Figure 1. YTHDF2 knockdown regulates the expression of inflammatory genes.

A. Immunoblot analysis of the protein levels of YTHDF2 and COX-2 in A431 cells with or without YTHDF2 knockdown.

B. qPCR analysis of the mRNA levels of IL-1β, IL-6, TNF-α, GM-CSF, IL-8, MMP-9, and VEGF in A431 with or without YTHDF2 knockdown. β-actin is used as the housekeeping gene.

C. qPCR analysis of the mRNA levels of GM-CSF, IL-16, IL-1α, IL-8, TGF-β, TNF-α, VEGF, COX-2, IL-1β, and IL-6 in HaCaT cells with or without YTHDF2 knockdown. β-actin is used as the housekeeping gene.

D. Immunofluorescence staining (and quantification) of COX-2 (red) as well as pan Cytokeratin (Green) in WT and DF2 cKO mouse skin following UVB exposure as in Figure 1C. DAPI is used as a nuclear counterstain.

E. qPCR analysis of the mRNA stability of TNF-α, IL-8, TGF-β, VEGF, COX-2, GM-CSF, IL-16, IL-1α, IL-1β, and IL-6 in HaCaT cells with or without YTHDF2 knockdown. β-actin is used as the housekeeping gene.

Statistical analyses were conducted using two-tailed unpaired Student's t-test (B, C, E). Mean ± SE are shown; n = 4 biologically independent samples (B, C, E). ns, not significant ($P > 0.05$). All experiments were conducted using biologically independent samples.

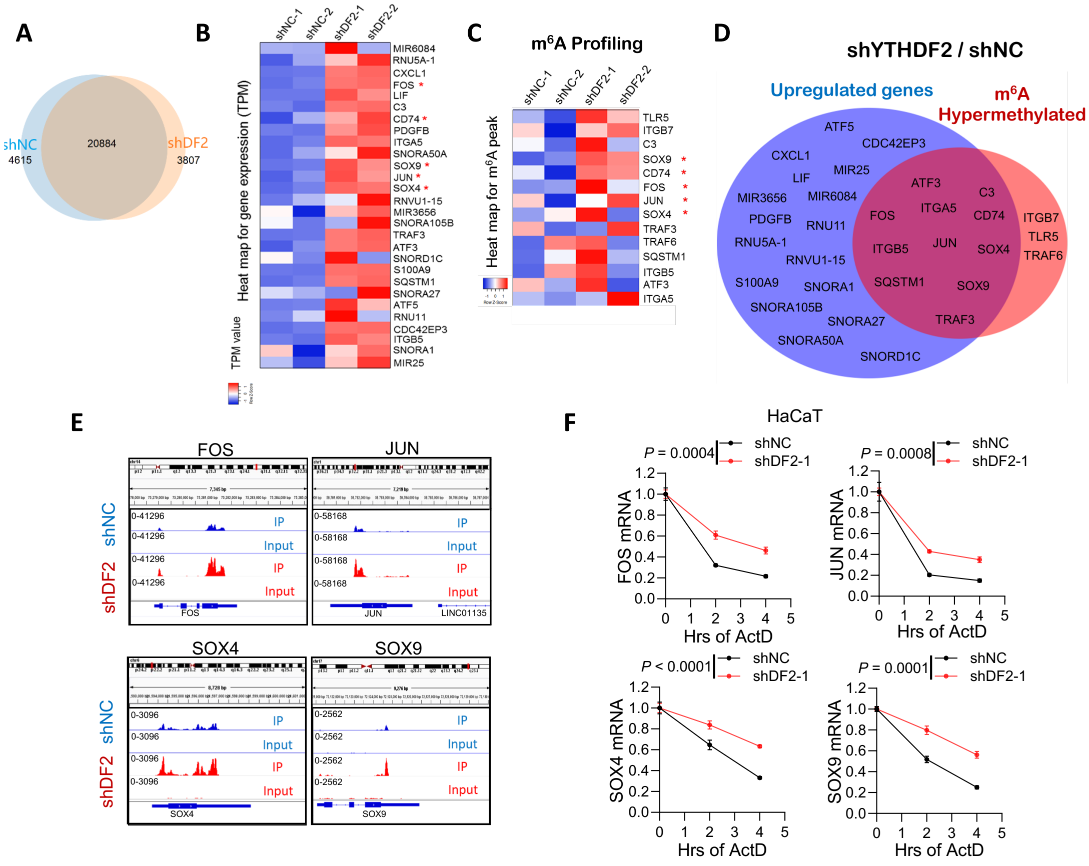

Supplementary Figure S7. Related to Figure 2. m^{6}A-seq analysis identifies potential mRNA targets for YTHDF2.
A. Venn diagram showing the overlap between control and YTHDF2 knockdown HaCaT cells in gene expression.
B. Heatmap showing the genes with altered expression in HaCaT cells by YTHDF2 knockdown.
C. Heatmap showing the genes with altered m^{6}A peak enrichment in HaCaT cells by YTHDF2 knockdown.
D. Venn diagram showing the overlap between the top 28 upregulated genes and the top 14 m^{6}A-hypermethylated genes.
E. Distribution of m^{6}A peaks across the transcripts of FOS, JUN, SOX4, or SOX9.
F. qPCR analysis of the mRNA stability of FOS, JUN, SOX4, or SOX9 in HaCaT cells with or without YTHDF2 knockdown. HPRT1 is used as the housekeeping gene.
Statistical analyses were conducted using two-tailed unpaired Student's t-test (F). Mean ± SE are shown; n = 4 biologically independent samples (F). ns, not significant ($P > 0.05$).

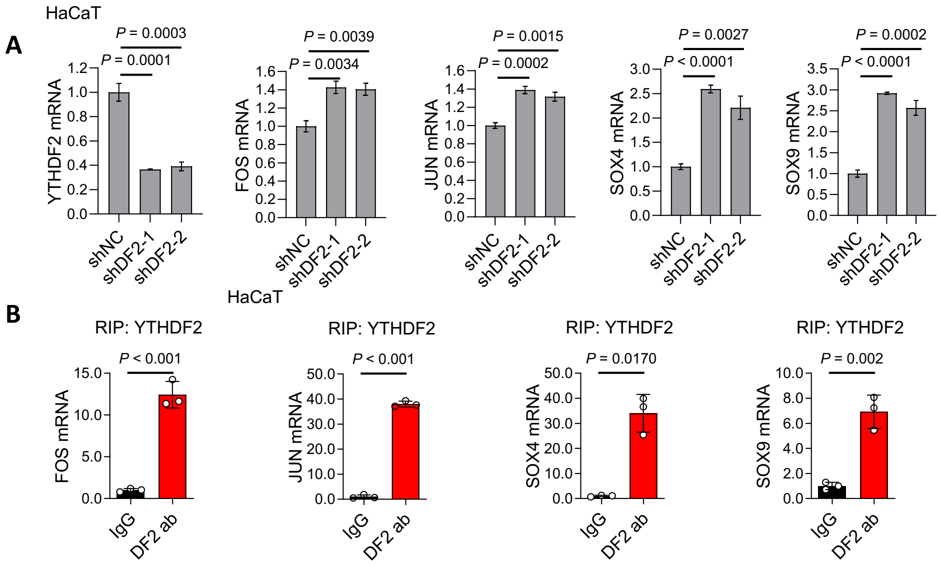

Supplementary Figure S8. Related to Figure 2. YTHDF2 regulates the expression of FOS, JUN, SOX4, and SOX9.

A. qPCR analysis of the mRNA levels for YTHDF2, FOS, JUN, SOX4, or SOX9 in HaCaT cells with or without YTHDF2 knockdown. HPRT1 is used as the housekeeping gene.
B. RIP analysis showing the interaction between YTHDF2 and the FOS, JUN, SOX4, or SOX9 mRNA in HaCaT cells.
Statistical analyses were conducted using two-tailed unpaired Student's t-test (A, B). Mean ± SE are shown; n = 4 biologically independent samples (A, B). ns, not significant (P > 0.05).

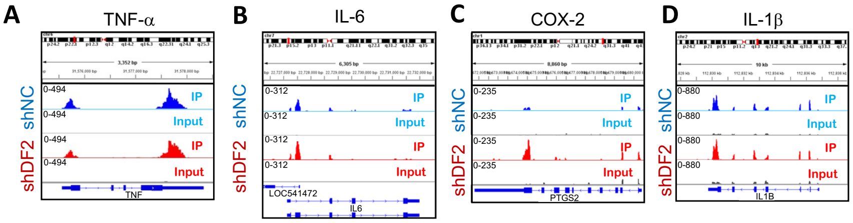

Supplementary Figure S9. Related to Figure 2. Distribution of m^{6}A peaks for inflammatory genes.
A-D. Distribution of m^{6}A peaks across the transcripts for TNF-α, IL-6, COX-2, and IL-1β.

A

Top 3 Non-coding snRNAs *Upregulated by UVB*	Number	Identified Proteins (RNA Binding Proteins)	Accession Number	MW (kDa)	Total Spectrum Count (Probability)		U6-associated RNPs
					Ctrl IgG IP	YTHDF2 IP	
**U5 snRNA**	1	116 kDa U5 small nuclear ribonucleoprotein component OS=Homo sapiens OX=9606 GN=EFTUD2 PE=1 SV=1	sp|Q15029|U5S1_HUMAN	109	0	36 (100%)	
	2	U4/U6.U5 tri-snRNP-associated protein 1 OS=Homo sapiens OX=9606 GN=SART1 PE=1 SV=1	sp|O43290|SNUT1_HUMAN	90	0	4 (100%)	*
	3	U4/U6.U5 tri-snRNP-associated protein 2 OS=Homo sapiens OX=9606 GN=USP39 PE=1 SV=2	sp|Q53GS9|SNUT2_HUMAN	65	0	3 (100%)	*
	4	U5 small nuclear ribonucleoprotein 40 kDa protein OS=Homo sapiens OX=9606 GN=SNRNP40 PE=1 SV=1	sp|Q96DI7|SNR40_HUMAN	39	5 (100%)	7 (100%)	
	5	U5 small nuclear ribonucleoprotein 200 kDa helicase OS=Homo sapiens OX=9606 GN=SNRNP200 PE=1 SV=2	sp|O75643|U520_HUMAN	245	48 (100%)	72 (100%)	
**U4 snRNA**	1	U4/U6.U5 tri-snRNP-associated protein 1 OS=Homo sapiens OX=9606 GN=SART1 PE=1 SV=1	sp|O43290|SNUT1_HUMAN	90	0	4 (100%)	*
	2	U4/U6 small nuclear ribonucleoprotein Prp3 OS=Homo sapiens OX=9606 GN=PRPF3 PE=1 SV=2	sp|O43395|PRPF3_HUMAN	78	0	6 (100%)	*
	3	U4/U6.U5 tri-snRNP-associated protein 2 OS=Homo sapiens OX=9606 GN=USP39 PE=1 SV=2	sp|Q53GS9|SNUT2_HUMAN	65	0	3 (100%)	*
	4	U4/U6 small nuclear ribonucleoprotein Prp4 OS=Homo sapiens OX=9606 GN=PRPF4 PE=1 SV=2	sp|O43172|PRP4_HUMAN	58	0	5 (100%)	*
**U6 snRNA**	1	U4/U6 small nuclear ribonucleoprotein Prp3 OS=Homo sapiens OX=9606 GN=PRPF3 PE=1 SV=2	sp|O43395|PRPF3_HUMAN	78	0	6 (100%)	*
	2	U4/U6 small nuclear ribonucleoprotein Prp4 OS=Homo sapiens OX=9606 GN=PRPF4 PE=1 SV=2	sp|O43172|PRP4_HUMAN	58	0	5 (100%)	*
	3	U4/U6.U5 tri-snRNP-associated protein 1 OS=Homo sapiens OX=9606 GN=SART1 PE=1 SV=1	sp|O43290|SNUT1_HUMAN	90	0	4 (100%)	*
	4	U4/U6.U5 tri-snRNP-associated protein 2 OS=Homo sapiens OX=9606 GN=USP39 PE=1 SV=2	sp|Q53GS9|SNUT2_HUMAN	65	0	3 (100%)	*
	5	U6 snRNA-associated Sm-like protein LSm2 OS=Homo sapiens OX=9606 GN=LSM2 PE=1 SV=1	sp|Q9Y333|LSM2_HUMAN	11	0	3 (100%)	*
	6	U6 snRNA-associated Sm-like protein LSm4 OS=Homo sapiens OX=9606 GN=LSM4 PE=1 SV=1	sp|Q9Y4Z0|LSM4_HUMAN	15	0	9 (100%)	*
	7	U6 snRNA-associated Sm-like protein LSm7 OS=Homo sapiens OX=9606 GN=LSM7 PE=1 SV=1	sp|Q9UK45|LSM7_HUMAN	12	2 (100%)	3 (100%)	*
	8	U6 snRNA-associated Sm-like protein LSm3 OS=Homo sapiens OX=9606 GN=LSM3 PE=1 SV=2	sp|P62310|LSM3_HUMAN	12	2 (100%)	4 (100%)	*
	9	U6 snRNA-associated Sm-like protein LSm8 OS=Homo sapiens OX=9606 GN=LSM8 PE=1 SV=3	sp|O95777|LSM8_HUMAN	10	4 (100%)	7 (100%)	*
	10	U6 snRNA-associated Sm-like protein LSm6 OS=Homo sapiens OX=9606 GN=LSM6 PE=1 SV=1	sp|P62312|LSM6_HUMAN	9	6 (100%)	12 (100%)	*

B

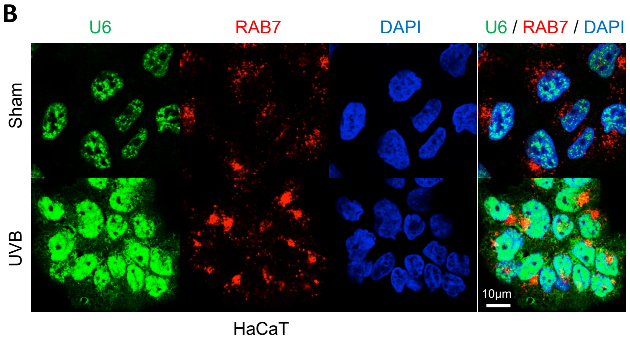

C

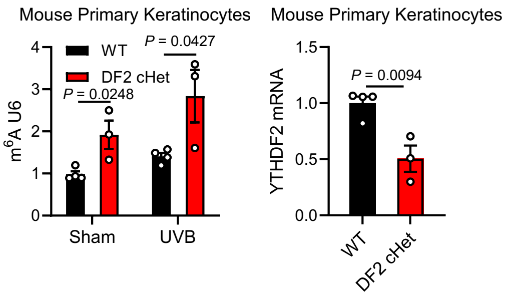

D

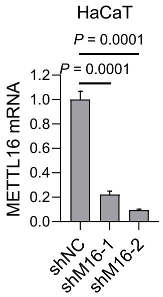

Supplementary Figure S10. Related to Figure 2. YTHDF2 association with U6 snRNA.
A. List of YTHDF2-interacting proteins associated with U5, U4, and U6 SNPs. Protein Threshold: 1% FDR (p-value < 0.01); Min # Peptide: 2; Peptide Threshold: 1% FDR (p-value < 0.01). U5, U4, and U6 snRNAs are shown to be upregulated by UVB irradiation in previous studies (Bernard et al., 2012).
B. FISH analysis of U6 snRNA and immunofluorescence analysis of Rab7 in HaCaT cells at 6 h post- sham or UVB irradiation (20 mJ/cm^2).
C. m^{6}A qPCR analysis of m^{6}A enrichment in U6 snRNA in mouse primary keratinocytes from mice with or without skin-conditional heterozygous YTHDF2 deletion (DF2 cHet) (Left) and qPCR analysis confirming YTHDF2 knockdown for left panel (Right). GAPDH is used as the housekeeping gene.
D. qPCR analysis confirming METTL16 knockdown for Fig. 2P-R. HPRT1 is used as the housekeeping gene.
Statistical analyses were conducted using two-tailed unpaired Student's t-test (C, D). Mean ± SE are shown; n = 4 (C, WT; D); n = 3 (C, DF2 cHet). ns, not significant (P > 0.05). All experiments were conducted using biologically independent samples.

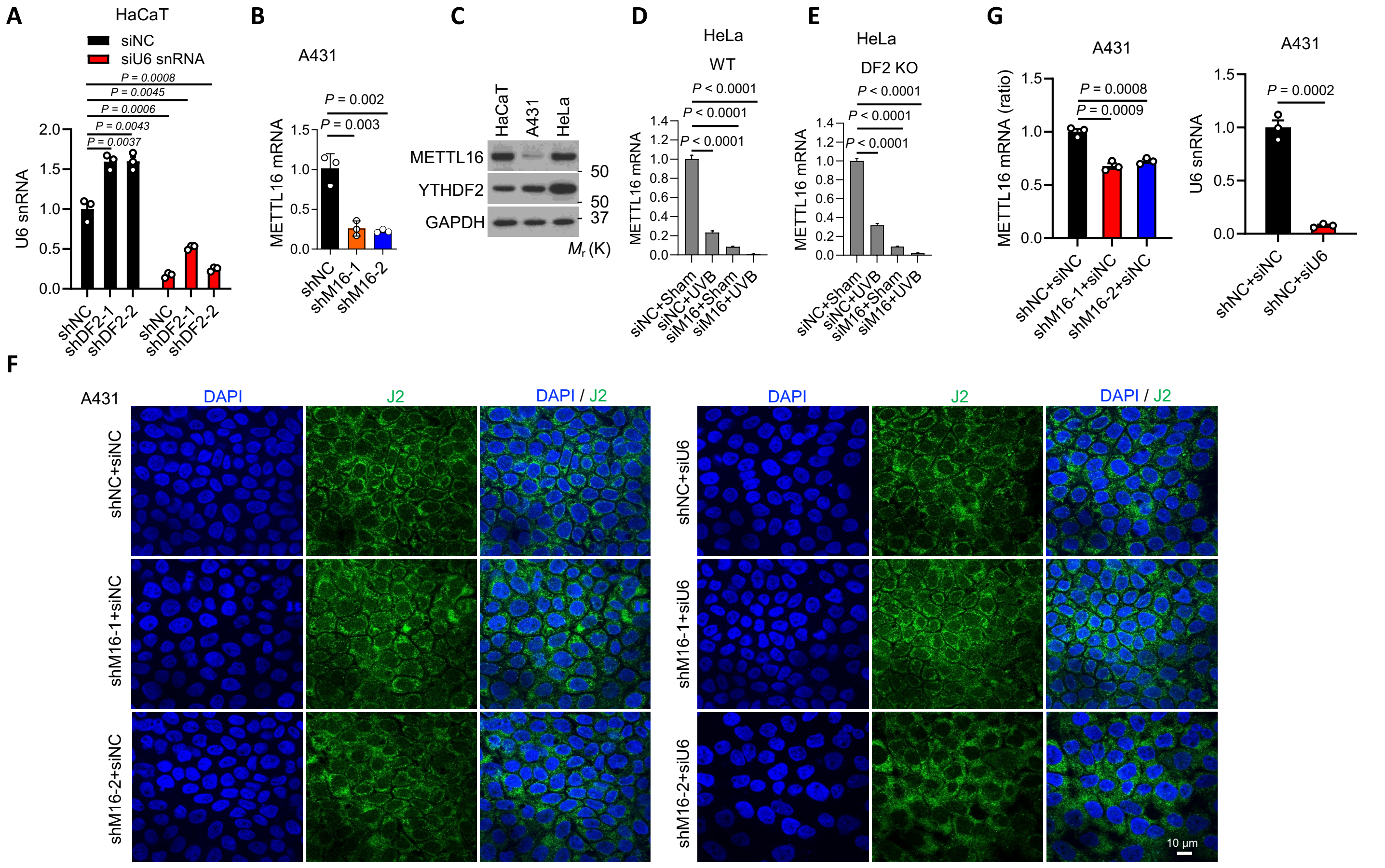

Supplementary Figure S11. Related to Figure 3. YTHDF2 controls inflammatory gene expression through U6 m^{6}A methylation.

A. qPCR analysis confirming U6 snRNA knockdown in HaCaT cells.

B. qPCR analysis confirming METTL16 knockdown in A431 cells.

C. Immunoblot analysis in HaCaT, A431, and HeLa cells.

D-E. qPCR analysis confirming METTL16 knockdown in WT and DF2 KO Hela cells treated with or without UVB irradiation.

F. Immunofluorescence of dsRNA using the anti-J2 antibody in A431 cells with or without METTL16 knockdown, U6 knockdown, or the combination.

G. qPCR analysis confirming METTL16 knockdown or U6 knockdown in A431 cells.

The housekeeping genes used are 18S rRNA (A, G-U6), HPRT1 (B, D-E), and GAPDH (G-METTL16).

Statistical analyses were conducted using two-tailed unpaired Student's t-test (A, B, D–E, G). Mean ± SE are shown; n = 4 (D–E); n = 3 (A, G). Mean ± SD are shown; n = 3 (B). ns, not significant (P > 0.05). All experiments were conducted using biologically independent samples.

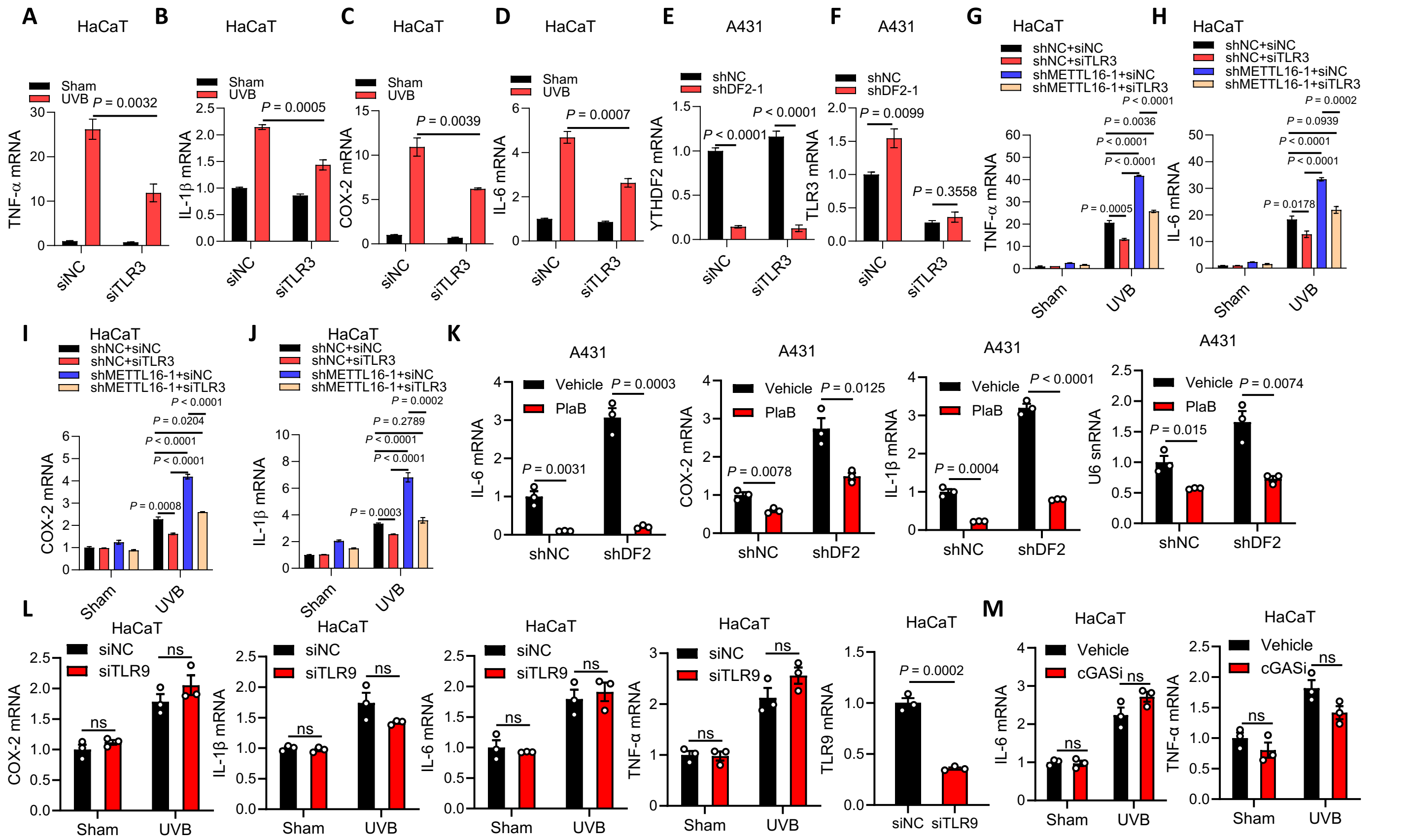

Supplementary Figure S12. Related to Figure 4. YTHDF2 interacts with m^{6}A U6 and thus inhibits m^{6}A U6 binding to TLR3.

A-D. qPCR analysis of the mRNA levels of TNF-α, IL-1β, COX-2, and IL-6 in siNC and siTLR3 HaCaT cells at 6 h post-sham and -UVB irradiation.

E-F. qPCR analysis confirming knockdown of YTHDF2 and TLR3 for Fig. 4E-G.

G-J. qPCR analysis of the mRNA level of TNF-α, IL-6, COX-2, and IL-1β in shNC and shM16 HaCaT cells transfected with siNC or siTLR3 at 6 h post-sham and -UVB irradiation.

K-M. qPCR analysis of the mRNA level of genes as indicated in in A431 cells treated with vehicle or PlaB (K), HaCaT cells with or without TLR9 knockdown at 6 h post-sham or UVB irradiation (L), and HaCaT treated with vehicle or cGAS inhibitor (cGASi) at 6 h post-sham or UVB irradiation (M).

The housekeeping genes used are HPRT1 (A-J), GAPDH (K except U6 snRNA, L, M), and 18S rRNA (K-U6 snRNA).

Statistical analyses were conducted using two-tailed unpaired Student's t-test (A–M). Mean ± SE are shown; n = 3–4 biologically independent samples. ns, not significant (P > 0.05). All experiments were conducted using biologically independent samples.

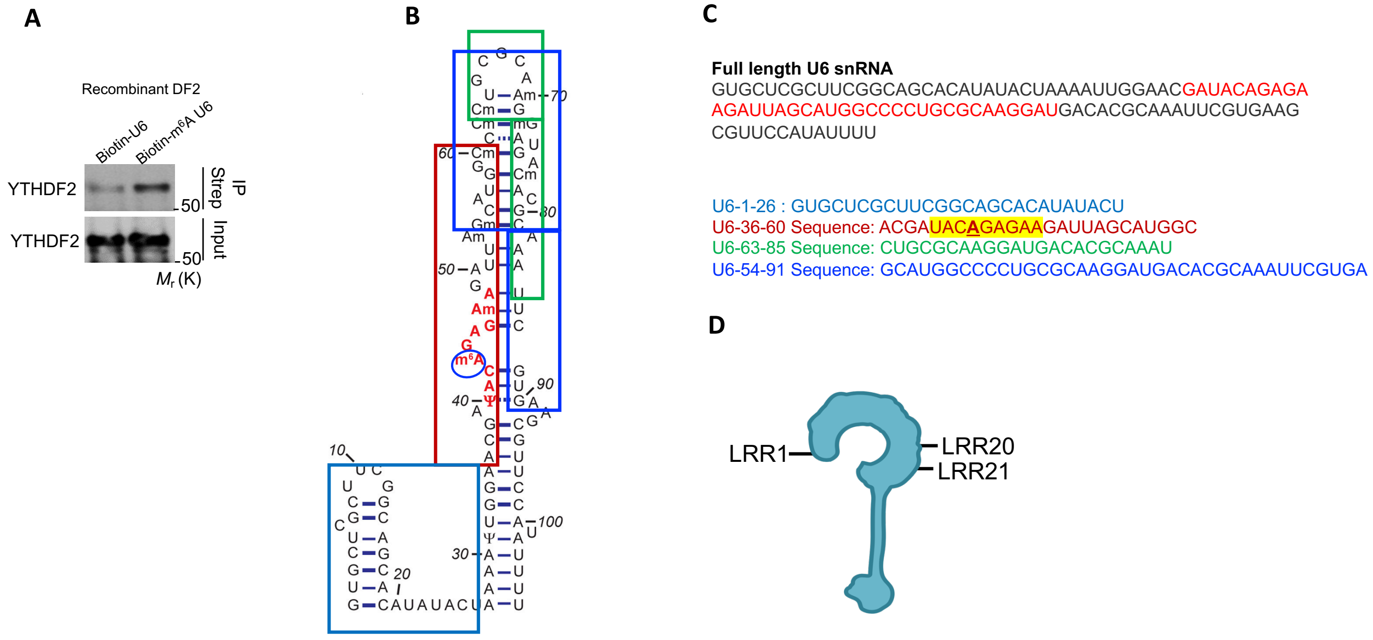

Supplementary Figure S13. Related to Figure 4. YTHDF2 interacts with m^{6}A U6 and thus inhibits m^{6}A U6 binding to TLR3.

A. Pulldown assay showing the interaction between U6 or m^{6}A U6 (biotin-labeled) with recombinant YTHDF2.
B. Structure of the free U6 snRNA. Adapted from previous reports (Mougin et al., 2002; Pendleton et al., 2017).
C. Sequence of U6 snRNA and truncated U6 oligos (1-26, 36-60, 54-91, 63-85).
D. Schematic of the LRR motifs of TLR3, created with Biorender.com.

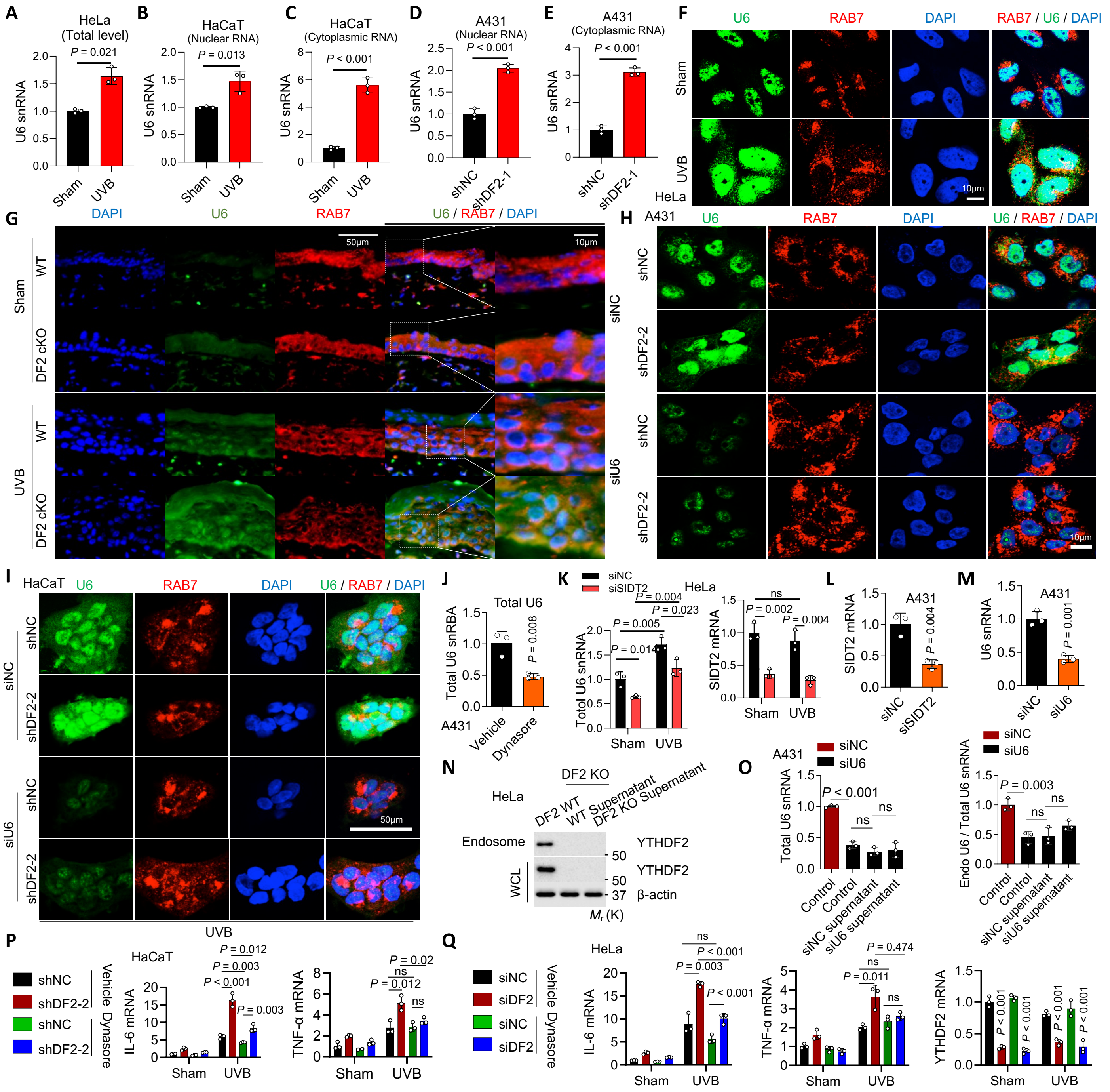

Supplementary Figure S14. Related to Figure 5. Both U6 and YTHDF2 are localized in endosomes.
A-C. qPCR analysis of nuclear/cytoplasmic U6 snRNA levels in HaCaT cells at 6 h post-sham or -UVB irradiation (20 mJ/cm^2).
D-E. qPCR analysis of nuclear/cytoplasmic U6 snRNA levels in A431 cells with or without YTHDF2 knockdown.
F-I. FISH analysis of U6 snRNA and immunofluorescence analysis of Rab7 in HeLa cells (F), mouse skin (G), A431 cells (H), and HaCaT cells (I). DAPI is used as a nuclear counterstain.
J. qPCR analysis of U6 snRNA levels in A431 cells with or without Dynasore.
K. qPCR analysis of U6 snRNA and SIDT2 mRNA levels in HeLa cells with or without siSIDT2 or in combination with or without UVB irradiation (30 mJ/cm^2) at 6 h.
L. qPCR analysis confirming SIDT2 knockdown in A431 cells.
M. qPCR analysis of U6 snRNA levels in A431 cells with or without siU6.

N. Immunoblot analysis of YTHDF2 in whole cell lysates (WCL) and endosomes in WT and DF2 KO HeLa cells treated with supernatant from WT or DF2 KO cells.
O. qPCR analysis of U6 snRNA in whole cell lysates (WCL) and endosomes in A431 cells with or without U6 knockdown treated with supernatant from A431 cells with or without U6 knockdown.
P-Q. qPCR analysis of the mRNA levels of IL-6 and TNF-α in HaCaT cells (P) and HeLa cells (Q) with or without YTHDF2 knockdown treated with vehicle or Dynasore at 6 h post-sham or -UVB irradiation (20 mJ/cm^2).
Statistical analyses were conducted using two-tailed unpaired Student's t-test (A–E, J–M, O–Q). Mean ± SD are shown; n = 3 (A–E, J–M, O-Q). ns, not significant (P > 0.05). All experiments were conducted using biologically independent samples.

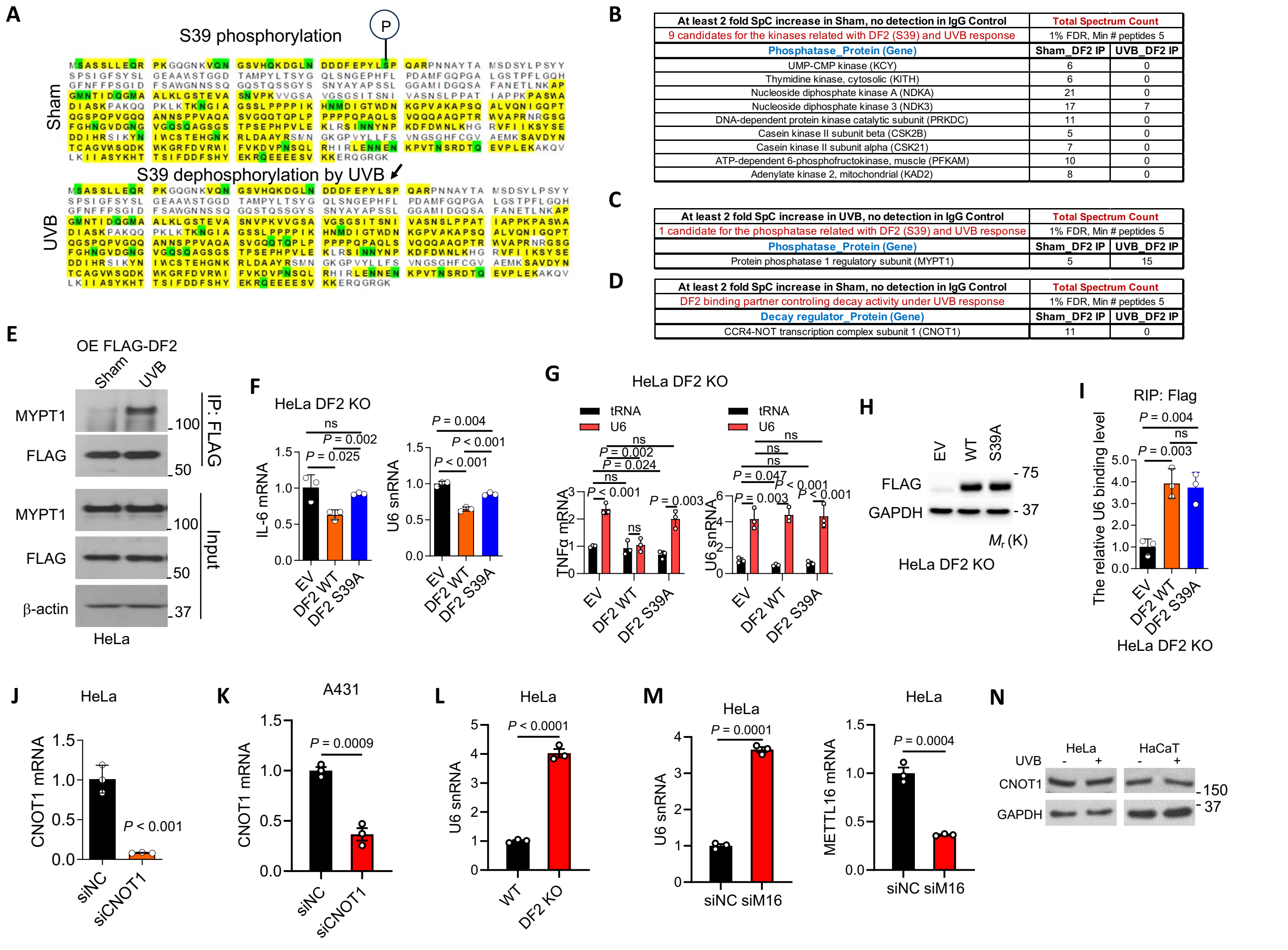

At least 2 fold SpC increase in Sham, no detection in IgG Control	Total Spectrum Count	
9 candidates for the kinases related with DF2 (S39) and UVB response	1% FDR, Min # peptides 5	
Phosphatase_Protein (Gene)	Sham_DF2 IP	UVB_DF2 IP
UMP-CMP kinase (KCY)	6	0
Thymidine kinase, cytosolic (KITH)	6	0
Nucleoside diphosphate kinase A (NDKA)	21	0
Nucleoside diphosphate kinase 3 (NDK3)	17	7
DNA-dependent protein kinase catalytic subunit (PRKDC)	11	0
Casein kinase II subunit beta (CSK2B)	5	0
Casein kinase II subunit alpha (CSK21)	7	0
ATP-dependent 6-phosphofructokinase, muscle (PFKAM)	10	0
Adenylate kinase 2, mitochondrial (KAD2)	8	0

At least 2 fold SpC increase in UVB, no detection in IgG Control	Total Spectrum Count	
1 candidate for the phosphatase related with DF2 (S39) and UVB response	1% FDR, Min # peptides 5	
Phosphatase_Protein (Gene)	Sham_DF2 IP	UVB_DF2 IP
Protein phosphatase 1 regulatory subunit (MYPT1)	5	15

At least 2 fold SpC increase in Sham, no detection in IgG Control	Total Spectrum Count	
DF2 binding partner controling decay activity under UVB response	1% FDR, Min # peptides 5	
Decay regulator_Protein (Gene)	Sham_DF2 IP	UVB_DF2 IP
CCR4-NOT transcription complex subunit 1 (CNOT1)	11	0

Supplementary Figure S15. Related to Figure 6. YTHDF2 phosphorylation is inhibited by UVB irradiation and plays an important role in U6 decay and endosomal localization.

A. Illustration of UVB-induced YTHDF2 S39 dephosphorylation in HaCaT cells at 1 h post-sham or -UVB irradiation (20 mJ/cm^2).

B-D. List of kinases, phosphatases, and proteins involved in RNA decay from mass spectrometric analysis of YTHDF2 binding proteins in HaCaT Cells at 1h post-sham or -UVB irradiation (20 mJ/cm^2).

E. Co-IP showing the effect of UVB irradiation on the interaction between MYPT1 and YTHDF2 in HeLa cells.

F. qPCR analysis of IL-6 mRNA and U6 snRNA levels in HeLa DF2 KO cells transfected with or without YTHDF2 WT (DF2 WT) and DF2 S39A.

G. qPCR analysis of TNF-α mRNA and U6 snRNA levels in HeLa DF2 KO cells transfected with or without YTHDF2 WT (DF2 WT) and DF2 S39A in combination with tRNA or U6.

H. Immunoblot analysis of YTHDF2 (FLAG) in HeLa DF2 KO cells transfected with or without YTHDF2 WT (DF2 WT) and DF2 S39A.

I. RIP analysis showing the interaction between U6 and YTHDF2 WT (DF2 WT) and DF2 S39A in HeLa DF2 KO cells.

J-K. qPCR analysis confirming CNOT1 knockdown in HeLa cells (P) and A431 cells (Q).

L-M. qPCR analysis of U6 snRNA level in HeLa cells with or without YTHDF2 deletion (R) and U6 snRNA level and METTL16 mRNA level in HeLa cells with or without METTL16 knockdown (S).

N. Immunoblot analysis of CNOT1 in HeLa and HaCaT cells at 1 h post-sham or -UVB irradiation (20 mJ/cm^2).

The housekeeping genes used are 18S rRNA (F-U6, G-U6, L, M-U6) and GAPDH (F-IL-6, G-TNF-α, J, K, M-METTL16).

Statistical analyses were conducted using two-tailed unpaired Student's t-test (F–G, I–M). Mean ± SD are shown; n = 3 (F–G, I). Mean ± SE are shown; n = 3 (J–M). ns, not significant ($P > 0.05$). All experiments were conducted using biologically independent samples.

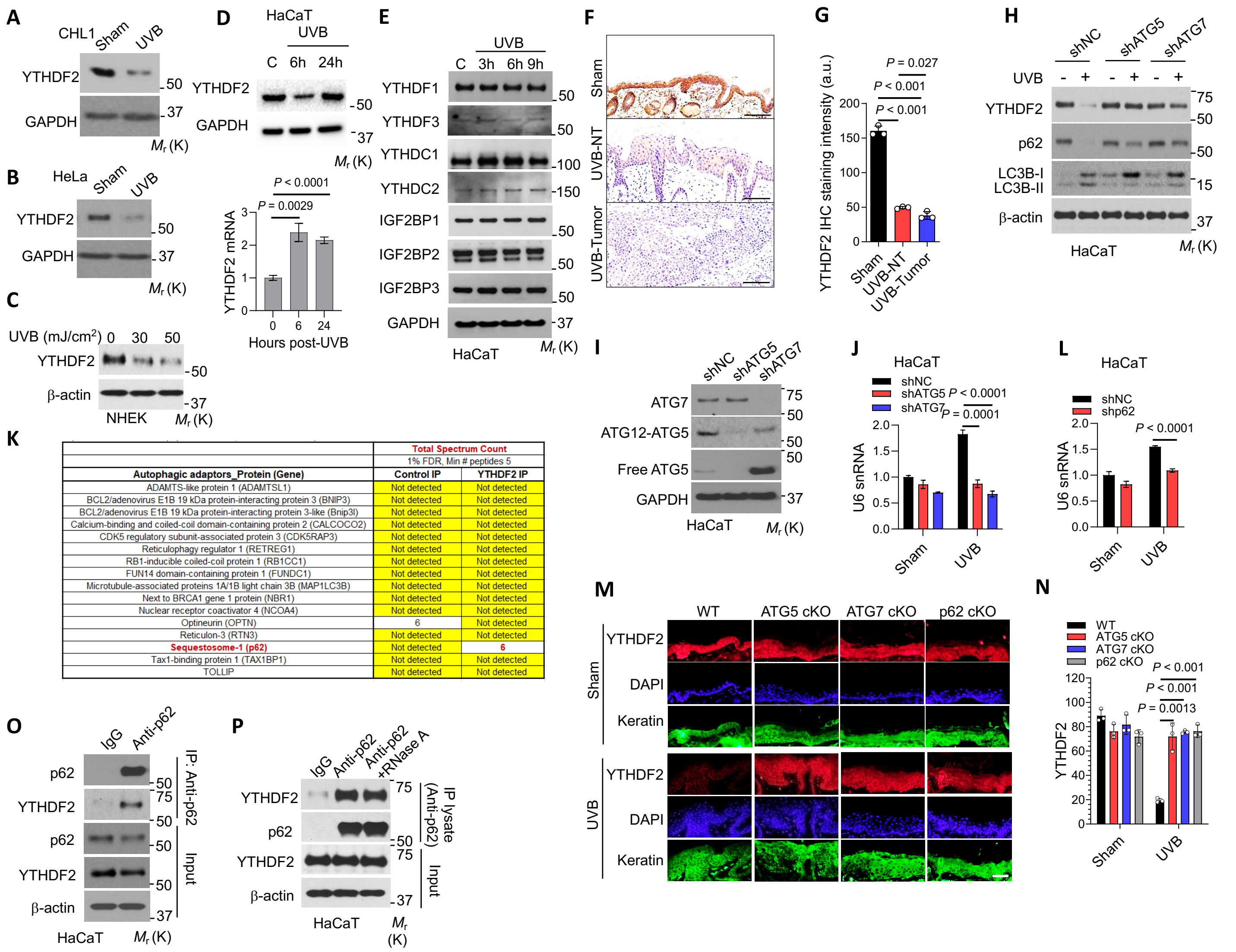

	Total Spectrum Count	
	1% FDR, Min # peptides 5	
Autophagic adaptors_Protein (Gene)	Control IP	YTHDF2 IP
ADAMTS-like protein 1 (ADAMTSL1)	Not detected	Not detected
BCL2/adenovirus E1B 19 kDa protein-interacting protein 3 (BNIP3)	Not detected	Not detected
BCL2/adenovirus E1B 19 kDa protein-interacting protein 3-like (Bnip3l)	Not detected	Not detected
Calcium-binding and coiled-coil domain-containing protein 2 (CALCOCO2)	Not detected	Not detected
CDK5 regulatory subunit-associated protein 3 (CDK5RAP3)	Not detected	Not detected
Reticulophagy regulator 1 (RETREG1)	Not detected	Not detected
RB1-inducible coiled-coil protein 1 (RB1CC1)	Not detected	Not detected
FUN14 domain-containing protein 1 (FUNDC1)	Not detected	Not detected
Microtubule-associated proteins 1A/1B light chain 3B (MAP1LC3B)	Not detected	Not detected
Next to BRCA1 gene 1 protein (NBR1)	Not detected	Not detected
Nuclear receptor coactivator 4 (NCOA4)	Not detected	Not detected
Optineurin (OPTN)	6	Not detected
Reticulon-3 (RTN3)	Not detected	Not detected
**Sequestosome-1 (p62)**	Not detected	**6**
Tax1-binding protein 1 (TAX1BP1)	Not detected	Not detected
TOLLIP	Not detected	Not detected

Supplementary Figure S16. Related to Figure 6. YTHDF2 is down-regulated by UVB irradiation.
A-B. Immunoblot analysis of YTHDF2 in CHL-1 cells (A) or HeLa cells (B) at 3 h post-sham or -UVB (30 mJ/cm^2).
C. Immunoblot analysis of YTHDF2 at 6 h post-sham or -UVB irradiation in NHEK cells.
D. Immunoblot analysis of YTHDF2 and qPCR analysis of YTHDF2 mRNA levels over a time course following sham or UVB (20 mJ/cm^2) irradiation.
E. Immunoblot analysis of other m^{6}A readers over a time course post-sham or -UVB irradiation in HaCaT cells.
F. Immunohistochemical analysis of the YTHDF2 protein levels in sham, UVB-exposed non-tumor (NT) skin, and UVB-induced tumor. Scale bar, 100 μm.
G. Quantification of YTHDF2 IHC staining intensity was performed using ImageJ (NIH) by separating RGB color channels and measuring signal intensity in the DAB-enriched channel.
H. Immunoblot analysis of YTHDF2, p62, and LC3B-I/II in HaCaT cells stably infected with shNC, shATG5, or shATG7 at 6 h post-sham or UVB irradiation (20 mJ/cm^2).
I. Immunoblot analysis of ATG7 and ATG5 in HaCaT cells.
J. qPCR analysis of U6 snRNA levels in HaCaT cells with or without knockdown of ATG5 and ATG7 or in combination with UVB irradiation (20 mJ/cm^2) at 6 h.
K. List of autophagy receptors from mass spectrometric analysis of YTHDF2 binding proteins.
L. qPCR analysis of U6 snRNA levels in HaCaT cells with or without p62 knockdown or in combination with UVB irradiation (20 mJ/cm^2) at 6 h.
M. Immunofluorescence analysis of YTHDF2 (red), Keratin (green), and DAPI (blue) in wild-type (WT), ATG5 cKO, ATG7 cKO, or p62 cKO mice. Scale bar, 100 μm.
N. Quantification of YTHDF2 protein levels using Image J in Keratin-positive cells in M.

O. Co-immunoprecipitation (Co-IP) showing the interaction between endogenous p62 and YTHDF2 in HaCaT cells.
P. Co-IP showing the effect of RNase A on the interaction between p62 and YTHDF2 in HaCaT cells.
The housekeeping genes used are β-actin (D) and 18S rRNA (J, L).
Statistical analyses were conducted using two-tailed unpaired Student's t-test (G, J, L, N). Mean ± SE are shown; $n = 4$ (D). Mean ± SD are shown; $n = 3$ for each group (G, J, L, N). ns, not significant ($P > 0.05$). **, $P < 0.01$; ***, $P < 0.001$. All experiments were conducted using biologically independent samples.

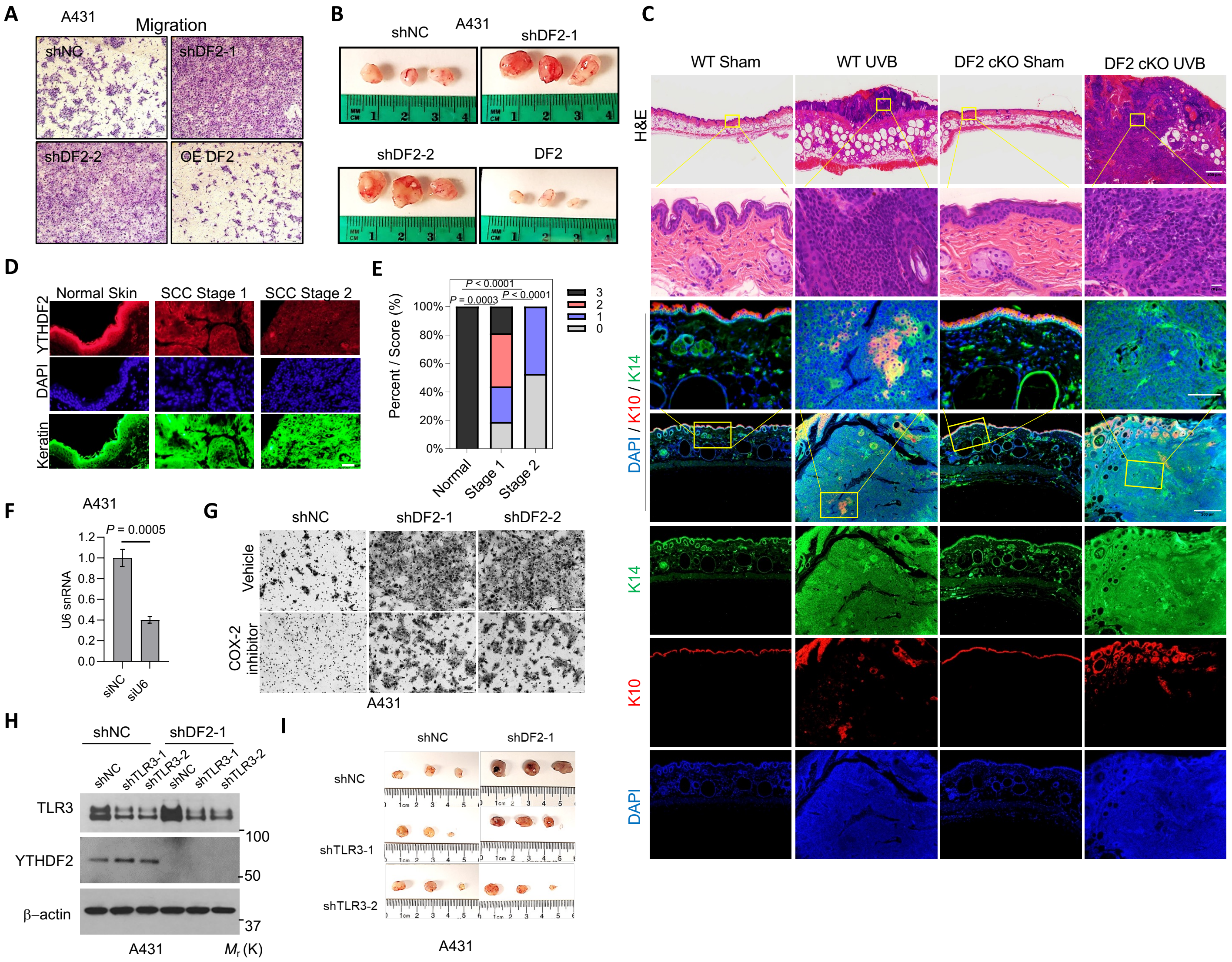

Supplementary Figure S17. Related to Figure 7. YTHDF2 controls tumorigenesis.
A. Representative images for migration assay for YTHDF2 knockdown and overexpression.
B. Representative images of tumors for Fig. 7E**.**
C. Histological analysis and immunofluorescence staining of K14 (green) and K10 (red) in mouse skin and tumor from WT and DF2 cKO mice. DAPI is used as a nuclear counterstain.
D. Immunofluorescence staining of YTHDF2 (red) in normal human skin and skin SCC tumors. Scale bar, 50 µm. Pan Keratin (green) is used as a keratinocyte marker.
E. Percentage of tumors (in stacked column format) for each score of YTHDF2. 0 (Negative), 1 (Weak), 2 (Medium), and 3 (Strong). Normal human skin (n=10), SCC stage 1 (n=17), SCC stage 2 (n = 38).
F. qPCR analysis confirming knockdown of U6 snRNA in A431 cells.
G. Representative images of migration assay for Fig. 7L. Scale bar, 100 µm.
H. Immunoblot analysis confirming knockdown of YTHDF2 and TLR3 for Fig. 7N-O.
I. Tumor images for Fig. 7O.
Statistical analyses were conducted using the Mann–Whitney U test (E) and two-tailed unpaired Student's t-test (F). Mean ± SE are shown; n = 4 (F). ns, not significant (P > 0.05)

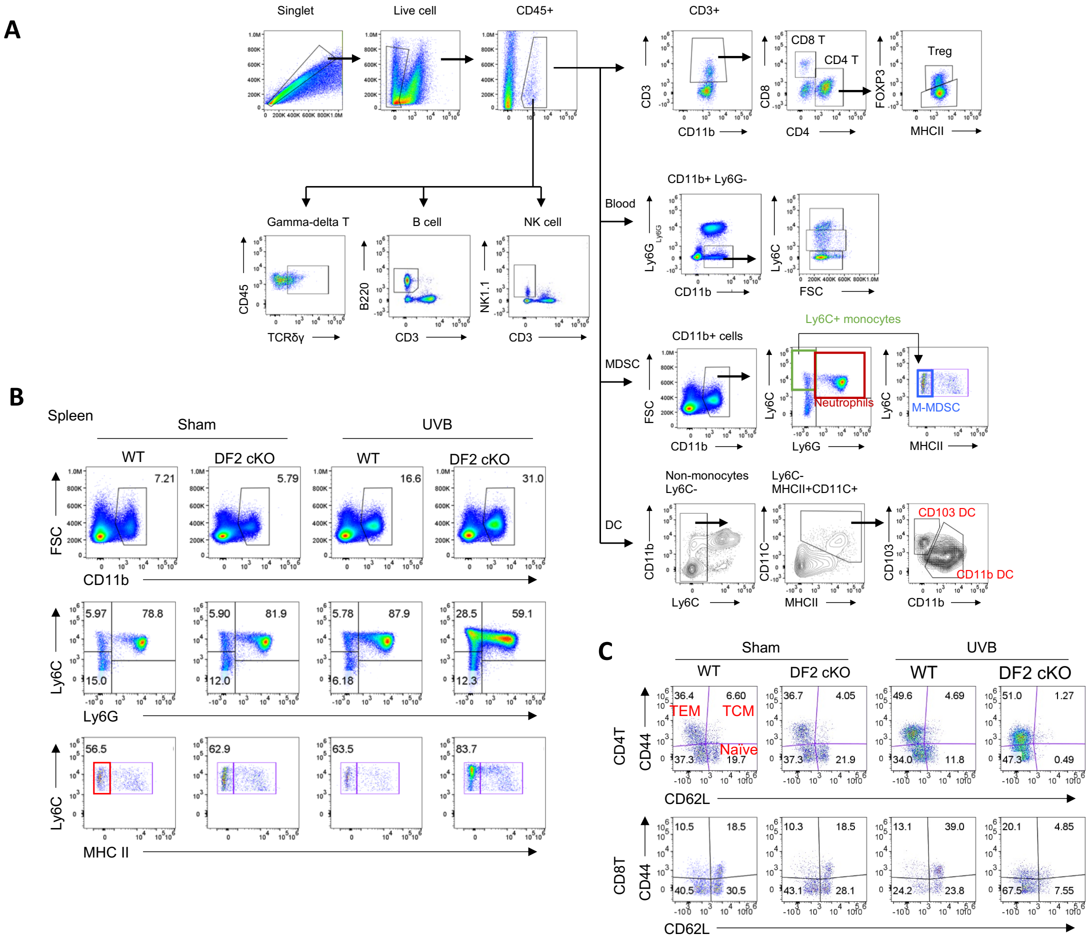

Supplementary Figure S18. Gating strategy for flow cytometric analysis of immune cell profiling.

Supplementary Table S2 Primers for cloning

Mutants of TLR3 and YTHDF2	Primer sequence (5'-3')
TLR3 LRR16-19 FORWARD	5' CACCTAGAAGTA GACATGTTGGAGGGTCTTGAG 3'
TLR3 LRR16-19 REVERSE	5' CTCCAACATGTC TACTTCTAGGTGGCCCAACCAA 3'
TLR3 LRR20 FORWARD	5' GAGGGTCTTGAG GGTGGTCCCATTTATTTCC 3'
TLR3 LRR20 REVERSE	5' AATGGGACCACC CTCAAGACCCTCCAACATGTC 3'
TLR3 LRR21 FORWARD	5' CCCATTTATTTC CCAGTTGAGGTCTTCAAGG 3'
TLR3 LRR21 REVERSE	5' CTCAACTGG GAAATAAATGGGACCACCAGG 3'
pCDNA3.0-HA-YTHDF2 FORWARD	5' ATGTACCCATACGATGTTCCAGATTACGCTATGTCGGCCAGCAGCCTCTT 3'
pCDNA3.0-HA-YTHDF2 REVERSE	5' AGCGTAATCTGGAACATCGTATGGGTACATGGATCCGAGCTCGATACCAA 3'
YTHDF2-S39A FORWARD	5' TTACTTGGCTCCACAGGCAAGGCCCAATAAT 3'
YTHDF2-S39A REVERSE	5' CCTGTGGAGCCAAGTAAGGTTCAAAATCATC 3'
YTHDF2-S39D-FORWARD	5' TTACTTGGATCCACAGGCAAGGCCCAATAAT 3'
YTHDF2-S39D REVERSE	5' CCTGTGGATCCAAGTAAGGTTCAAAATCATC 3'

Supplementary Table S3. Primers for qPCR analysis

Primers

GENE NAME	Primer Direction	Primer Sequence
YTHDF2	Forward	5' – TCT GGA AAA GGC TAA GCA GG - 3'
	Reverse	5' – CTT TTA TTT CCC ACG ACC TTG AC - 3'
TNF-α	Forward	5'- ACT TTG GAG TGA TCG GCC -3'
	Reverse	5'- GCT TGA GGG TTT GCT ACA AC -3'
IL-6	Forward	5'- CCA CTC ACC TCT TCA GAA CG -3'
	Reverse	5'- CAT CTT TGG AAG GTT CAG GTT G -3'
COX-2	Forward	5'- CCC TTG GGT GTC AAAGGT AA -3'
	Reverse	5'- GCC CTG GCT TAT GAT CTG TC -3'
IL-1β	Forward	5'- GCT TGG TGA TGT CTG GTC CAT -3'
	Reverse	5'- CAC CAC TTG TTG CTC CAT ATC CT -3'
GM-CSF	Forward	5'- CAC TGC TGC TGA GAT GAA TGA AA -3'
	Reverse	5'- GTC TGT AGG CAG GTC GGC TC -3'
IL-8	Forward	5'- ATG ACT TCC AAG CTG GCC GTG GCT -3'
	Reverse	5'- TCT CAG CCC TCT TCA AAA ACT TCT -3'
MMP-9	Forward	5'- TTG ACA GCG ACA AGA AGT GG -3'
	Reverse	5'- GCC ATT CAC GTC GTC CTT AT -3'

VEGF	Forward	5'- CTA CCT CCA CCA TGC CAA GT -3'
	Reverse	5'- GCA GTA GCT GCG CTG ATA GA -3'
IL-16	Forward	5'- GGC CTC ACA CGG TTT GAA G -3'
	Reverse	5'- CAA TCG TGA CAG GTC CAT CAG -3'
IL-1α	Forward	5'- GTT TAA GCC AAT CCA TCA CTG ATG -3'
	Reverse	5'- GAC CTA GGC TTG ATG ATT TCT TCC T -3'
FOS	Forward	5'- TTG TGA AGA CCA TGA CAG GAG -3'
	Reverse	5'- CCA TCT TAT TCC TTT CCC TTC GG -3'
JUN	Forward	5'- AGC CCA AAC TAA CCT CAC G -3'
	Reverse	5'- TGC TCT GTT TCA GGA TCT TGG -3'
SOX4	Forward	5'- AAA GAC AGC GAC AAG ATC CC -3'
	Reverse	5'- TTG CCG GAC TTC ACC TTC -3'
SOX9	Forward	5'- CAC AGC TCA CTC GAC CTT G -3'
	Reverse	5'- ACA CAA ATG TCC AAA GGG AAT TC -3'
CD74	Forward	5'- GGA AGA TCA GAA GCC AGT CAT G -3'
	Reverse	5'- AGG ATG GAA AAG CCT GTG TAC -3'
HPRT1	Forward	5'- TGC TGA GGA TTT GGA AAG GG -3'
	Reverse	5'- ACA GAG GGC TAC AAT GTG ATG -3'
U6 snRNA (#1) (Lou et al., 2015)	Forward	5'- GCT TCG GCA GCA CAT ATA CTA AAA T -3'
	Reverse	5'- CGC TTC ACG AAT TTG CGT GTC AT -3'
U6 snRNA (#2)	Forward	5'- CTC GCT TCG GCA GCA CAT ATA CT -3'
	Reverse	5'- ACG CTT CAC GAA TTT GCG TGT C -3'
METTL16	Forward	5'- TGA ATT CTG TCA AGG TCG GAC -3'
	Reverse	5'- GTT ATG GGT TTT CTC GGT TTC TC -3'
TLR3	Forward	5'- TCA ACT TTC TGA TAA AAC CTT TGC C -3'
	Reverse	5'- AGA TGA CAA GCC ATT ATG AGA CA -3'
GAPDH	Forward	5’ – AAT CCC ATC ACC ATC TTC CA - 3’
	Reverse	5’ – TGG ACT CCA CGA CGT ACT CA - 3’
GAPDH	Forward	5’ – ACA TCG CTC AGA CAC CAT G - 3’
	Reverse	5’ – TGT AGT TGA GGT CAA TGA AGG G - 3’
β-actin	Forward	5'- ACC TTC TAC AAT GAG CTG CG -3'
	Reverse	5'- CCT GGA TAG CAA CGT ACA TGG -3'
18S rRNA	Forward	5'- GGC CCT GTA ATT GGA ATG AGT C -3'
	Reverse	5'- CCA AGA TCC AAC TAC GAG CTT -3'
ATG5	Forward	5'- TCA GCC ACT GCA GAG GTG TTT -3'
	Reverse	5'- GGC TGC AGA TGG ACA GTT GCA -3'
ATG7	Forward	5'- TTT GCT ATC CTG CCC TCT -3'
	Reverse	5'- TGC CTC CTT TCT GGT TCT -3'
p62	Forward	5'- AAT CAG CTT CTG GTC CAT CG -3'
	Reverse	5'- TTC TTT TCC CTC CGT GCT C -3'
BCAT1	Forward	5'- GGG AGG GAA TTA CGG CTC AT -3'

	Reverse	5'- TGA TGT GCC AGG TCC AGA AT -3'
BCAT2	Forward	5'- CTG TGC TCA TTG GGA ACG AG -3'
	Reverse	5'- TAA CAC GGT GGG CCC ATA AT -3'
SIDT2	Forward	5'- CAC CTT CAA CCA TAC TGT GAC C -3'
	Reverse	5'- AAC ATC CCT CGC AGG ATT AGG -3'
MAT2A	Forward	5'- TCA GAA GAG TGA GAG AGA GCT AT -3'
	Reverse	5'- CCA TAG GCT GCA GTC CTC -3'
TLR9	Forward	5’-CCTTCGTGGTCTTCGACAAAAC-3’
	Reverse	5’-TTGTACACCCAGTCTGCCACTG-3’
MYPT1	Forward	5’-GCAGGTGTTACACGTTCAGCTTC-3’
	Reverse	5’-GATGTACTGGCTAGTCGTCTTGG-3’
mTNF-α	Forward	5’-GGTGCCTATGTCTCAGCCTCTT-3’
	Reverse	5’-GCCATAGAACTGATGAGAGGGAG-3’
mIL-6	Forward	5’-ACAAAGCCAGAGTCCTTCAGAGAG-3’
	Reverse	5’-TTGGATGGTCTTGGTCCTTAGCCA-3’
mIL-1β	Forward	5’-TGGACCTTCCAGGATGAGGACA-3’
	Reverse	5’-GTTCATCTCGGAGCCTGTAGTG-3’
mCOX-2 (ptgs2)	Forward	5’-GCGACATACTCAAGCAGGAGCA-3’
	Reverse	5’-AGTGGTAACCGCTCAGGTGTTG-3’
mGAPDH	Forward	5’-CATCACTGCCACCCAGAAGACTG-3’
	Reverse	5’-ATGCCAGTGAGCTTCCCGTTCAG-3’

Supplementary Table S4. Antibodies for IF staining

**Reagents or Resource**	**Source**	**Identifier**	Dilution
Alexa Fluor® 488 Donkey Anti-Guinea Pig IgG (H+L)	Jackson ImmunoResearch	706-545-148	1/100
Alexa Fluor® 488 Donkey Anti-Mouse IgG (H+L)	Jackson ImmunoResearch	715-545-150	1/100
Alexa Fluor® 594 Donkey Anti-Rabbit IgG (H+L)	Jackson ImmunoResearch	711-585-152	1/100
Pan-cytokeratin	Origene	BP5069	1/200
RAB7	Abcam	Ab50533	1/100
YTHDF2 (IHC and IF)	Proteintech	#247441-1-AP	1/500
YTHDF2 (Confocal)	Abcam	ab246514	1/200
dsRNA (IF)	SCIONS	10010200	1/150
Cytokeratin 14 (IF)	abcam	Ab7800	1/1000
Cytokeratin 10 (IF)	abcam	Ab76318	1/5000
COX-2 (IF)	Santa Cruz	sc-376861	1/50

Supplementary Table S5. Antibodies for immunoblotting

Reagents or Resource	Source	Identifier	Dilution
ATG5	Cell Signaling Technology	2630S	1/1000
ATG7	Cell Signaling Technology	8558S	1/1000
beta-actin	Santa Cruz	SC-47778	1/10000
COX-2	Santa Cruz	SC-19999	1/500
FLAG-Tag (DYKDDDDK)	Cell Signaling Technology	8146S	1/500
FLAG-Tag (DYKDDDDK)	Cell Signaling Technology	14793S	1/500
FLAG-Tag (DYKDDDDK)	Proteintech	20543-1-AP	1/2000
GAPDH	Santa Cruz	SC-47724	1/10000
HA-Tag	Cell Signaling Technology	3724S	1/2000
HA-Tag	Sigma	H3663	1/2000
IGF2BP1 (IMP1)	Cell Signaling Technology	8482S	1/1000
IGF2BP2 (IMP2)	Cell Signaling Technology	14672S	1/1000
IGF2BP3 (IMP3)	Cell Signaling Technology	57145S	1/1000
LC3B	Cell Signaling Technology	3868S	1/500
METTL16	Cell Signaling Technology	17676S	1/2000
p62	Sigma	P0067	1/2000
p62	Progen Biotechnik GmbH	GP62-C	1/10000
TLR3	Cell Signaling Technology	6961S	1/500
TLR3	Abcam	ab62566	1/500
TLR3	Abcam	ab13915	1/500
YTHDC1	Abcam	ab122340	1/1000
YTHDC2	Abcam	ab176846	1/1000
YTHDF1	Proteintech	66745-1-Ig	1/2000
YTHDF2	Abcam	ab220163	1/5000
YTHDF2	Proteintech	247441-1-AP	1/5000
YTHDF2	Cell Signaling Technology	80014S	1/2000
YTHDF3	Cell Signaling Technology	24206S	1/500
PARP1	Santa Cruz	sc-8007	1/500
RAB5	Abcam	ab18211	1/500
RAB7	Abcam	ab50533	1/1000
YTHDF2 pS39 Ab	Generated by ABclonal		1/1000
CNOT1	Proteintech	66507-1-Ig	1/500

Supplementary Table S6. Antibodies for flow cytometric analysis

Antibodies	Clone	Source	Identifier
FITC anti-mouse CD103 Antibody	2 E7	Biolegend	121420
FITC anti-mouse CD3 Antibody	17A2	Biolegend	100203
FOXP3 Monoclonal Antibody, PE	NRRF-30	Thermo fisher	12-4771-82
PerCP/Cy5.5 anti-mouse/human CD45R/B220 Antibody	RA3-6B2	Biolegend	103236
PerCP/Cyanine5.5 anti-mouse/human CD11b Antibody	101228	Biolegend	101228
PE/Cy7 anti-mouse CD279 (PD-1) Antibody	29F.1A12	Biolegend	109110
PE/Cyanine7 anti-mouse/human CD44 Antibody	IM7	Biolegend	103030
PE/Cyanine7 anti-mouse Ly-6C Antibody	HK1.4	Biolegend	128018
APC anti-mouse CD11C Antibody	N418	Biolegend	117310
APC anti-mouse CD62L Antibody	MEL-14	Biolegend	104412
Brilliant Violet 421™ anti-mouse I-A/I-E Antibody	M5/114.15.2	Biolegend	107632
Brilliant Violet 421™ anti-mouse NK-1.1 Antibody	PK136	Biolegend	100451
Brilliant Violet 510™ anti-mouse Ly-6G Antibody	1A8	Biolegend	127633
Brilliant Violet 605™ anti-mouse CD4 Antibody	GK1.5	Biolegend	108741
Brilliant Violet 650™ anti-mouse CD45 Antibody	30-F11	Biolegend	103151
Brilliant Violet 711™ anti-mouse CD8a Antibody	53-6.7	Biolegend	100759
APC Rat IgG2a, κ Isotype Ctrl Antibody	RTK2758	Biolegend	400512
PE/Dazzle™ 594 Armenian Hamster IgG Isotype Ctrl Antibody	HTK888	Biolegend	400951
PerCP/Cy5.5 Syrian Hamster IgG Isotype Ctrl Antibody	SHG-1	Biolegend	402027
FITC Armenian Hamster IgG Isotype Ctrl Antibody	HTK888	Biolegend	400906
PE Mouse IgG1, κ Isotype Ctrl (ICFC) Antibody	MOPC-21	Biolegend	400140
PE/Cyanine7 Rat IgG2b, κ Isotype Ctrl Antibody	RTK4530	Biolegend	400618

References

Bernard, J.J., Cowing-Zitron, C., Nakatsuji, T., Muehleisen, B., Muto, J., Borkowski, A.W., Martinez, L., Greidinger, E.L., Yu, B.D., and Gallo, R.L. (2012). Ultraviolet radiation damages self noncoding RNA and is detected by TLR3. Nat Med *18*, 1286-1290.

Lou, G., Ma, N., Xu, Y., Jiang, L., Yang, J., Wang, C., Jiao, Y., and Gao, X. (2015). Differential distribution of U6 (RNU6-1) expression in human carcinoma tissues demonstrates the requirement for caution in the internal control gene selection for microRNA quantification. Int J Mol Med *36*, 1400-1408.

Mougin, A., Gottschalk, A., Fabrizio, P., Luhrmann, R., and Branlant, C. (2002). Direct probing of RNA structure and RNA-protein interactions in purified HeLa cell's and yeast spliceosomal U4/U6.U5 tri-snRNP particles. J Mol Biol *317*, 631-649.

Pendleton, K.E., Chen, B., Liu, K., Hunter, O.V., Xie, Y., Tu, B.P., and Conrad, N.K. (2017). The U6 snRNA m(6)A Methyltransferase METTL16 Regulates SAM Synthetase Intron Retention. Cell *169*, 824-835 e814.
