# Supplementary material for: YTHDF2 regulates self non-coding RNA metabolism to control inflammation and tumorigenesis
Source: Nat Commun. 2025 Nov 12;16:9946. doi: 10.1038/s41467-025-64898-7 (PMC12612255; doi:10.1038/s41467-025-64898-7)
Supplement: Supplementary file 3 — Reporting Summary [file 41467_2025_64898_MOESM3_ESM.pdf]

nature portfolio

Corresponding author(s): Yu-Ying He

Last updated by author(s): Sep 19, 2025

Reporting Summary

Nature Portfolio wishes to improve the reproducibility of the work that we publish. This form provides structure for consistency and transparency in reporting. For further information on Nature Portfolio policies, see our Editorial Policies and the Editorial Policy Checklist.

Statistics

For all statistical analyses, confirm that the following items are present in the figure legend, table legend, main text, or Methods section.

n/a	Confirmed	
☐	☒	The exact sample size (*n*) for each experimental group/condition, given as a discrete number and unit of measurement
☐	☒	A statement on whether measurements were taken from distinct samples or whether the same sample was measured repeatedly
☐	☒	The statistical test(s) used AND whether they are one- or two-sided<br>*Only common tests should be described solely by name; describe more complex techniques in the Methods section.*
☒	☐	A description of all covariates tested
☒	☐	A description of any assumptions or corrections, such as tests of normality and adjustment for multiple comparisons
☐	☒	A full description of the statistical parameters including central tendency (e.g. means) or other basic estimates (e.g. regression coefficient) AND variation (e.g. standard deviation) or associated estimates of uncertainty (e.g. confidence intervals)
☐	☒	For null hypothesis testing, the test statistic (e.g. *F*, *t*, *r*) with confidence intervals, effect sizes, degrees of freedom and *P* value noted<br>*Give P values as exact values whenever suitable.*
☒	☐	For Bayesian analysis, information on the choice of priors and Markov chain Monte Carlo settings
☒	☐	For hierarchical and complex designs, identification of the appropriate level for tests and full reporting of outcomes
☒	☐	Estimates of effect sizes (e.g. Cohen's *d*, Pearson's *r*), indicating how they were calculated

Our web collection on statistics for biologists contains articles on many of the points above.

Software and code

Policy information about availability of computer code

Data collection	For Immunofluorescence analyasis: Cell Sens standard Ver.1.17<br>For mRNA level analysis : Bio-Rad CFX Manager
Data analysis	For Statistical analysis: Prism 10, Image J (National Institutes of Health)<br>Sequencing analysis: the adapters were removed by using cutadapt for m6A-seq, reads were aligned to the reference genome (hg38) using Tophat v2.0.14 with parameter -g 1 --library-type=fr-firststrand. RefSeq Gene structure annotations were downloaded from UCSC Table Browser.<br>Flow cytometric analysis was performed on a Fortessa 4-15 (BD Biosciences) and Attune NxT (Thermo Fisher Scientific) with Flowjo V10.6.1 used for analysis.<br>Gene ontology (GO) enrichment and Kyoto Encyclopedia of Genes and Genomes (KEGG) pathway enrichment analyses were performed using Metascape (https://metascape.org/), a web-based portal designed to provide a comprehensive gene list annotation and analysis resource, as described previously 57. Quantitative Venn diagrams were generated using the following web tool: https://www.deepvenn.com/.

For manuscripts utilizing custom algorithms or software that are central to the research but not yet described in published literature, software must be made available to editors and reviewers. We strongly encourage code deposition in a community repository (e.g. GitHub). See the Nature Portfolio guidelines for submitting code & software for further information.

Data

Policy information about availability of data

All manuscripts must include a data availability statement. This statement should provide the following information, where applicable:

- Accession codes, unique identifiers, or web links for publicly available datasets
- A description of any restrictions on data availability
- For clinical datasets or third party data, please ensure that the statement adheres to our policy

m6A IP sequencing and RNA sequencing data have been deposited in the GEO repository under accession code GSE145925 [https://www.ncbi.nlm.nih.gov/geo/query/acc.cgi?acc=GSE145925].
Mass spectrometry data have been deposited in the PRIDE repository under accession code PXD059422 [http://proteomecentral.proteomexchange.org/cgi/GetDataset?ID=PXD059422].

Research involving human participants, their data, or biological material

Policy information about studies with human participants or human data. See also policy information about sex, gender (identity/presentation), and sexual orientation and race, ethnicity and racism.

Reporting on sex and gender	N/A (no human research participants)
Reporting on race, ethnicity, or other socially relevant groupings	N/A (no human research participants)
Population characteristics	N/A (no human research participants)
Recruitment	N/A (no human research participants)
Ethics oversight	N/A (no human research participants)

Note that full information on the approval of the study protocol must also be provided in the manuscript.

Field-specific reporting

Please select the one below that is the best fit for your research. If you are not sure, read the appropriate sections before making your selection.

☒ Life sciences ☐ Behavioural & social sciences ☐ Ecological, evolutionary & environmental sciences

For a reference copy of the document with all sections, see nature.com/documents/nr-reporting-summary-flat.pdf

Life sciences study design

All studies must disclose on these points even when the disclosure is negative.

Sample size	Generally no statistical analysis was performed to predetermine the sample size. Sample size and number of animals are selected based on our previous experiences of carrying out similar experiments and published work. Sample size and number of independent experiments are clearly stated in the figure legend or in the Methods section. Three to more independent replicates were used to perform statistical analyses.
Data exclusions	No data were excluded from analysis.
Replication	Experiments were repeated two to three times independently. Replication were described in the figure legends.
Randomization	For the xenograft model, animals were randomly assigned into groups receiving various cell line injections. Randomization (formal or otherwise) was not relevant for other data included in the manuscript.
Blinding	For immunofluorescence (IF) experiment, blind staining and blind analysis were carried out. For other experiments, the investigators were not blinded to group allocation during data collection and/or analysis.

Reporting for specific materials, systems and methods

We require information from authors about some types of materials, experimental systems and methods used in many studies. Here, indicate whether each material, system or method listed is relevant to your study. If you are not sure if a list item applies to your research, read the appropriate section before selecting a response.

Materials & experimental systems

n/a	Involved in the study
	☒ Antibodies
	☒ Eukaryotic cell lines
☒	Palaeontology and archaeology
	☒ Animals and other organisms
☒	Clinical data
☒	Dual use research of concern
☒	Plants

Methods

n/a	Involved in the study
☒	ChIP-seq
	☒ Flow cytometry
☒	MRI-based neuroimaging

Antibodies

Antibodies used

Western blot/IP
ATG5, Cell Signaling Technology, #2630S
ATG7, Cell Signaling Technology, #8558S
beta-actin, Santa Cruz, SC-47778
COX-2, Santa Cruz, SC-19999
Flag-Tag (DYKDDDDK), Cell Signaling Technology, 8146S
Flag-Tag (DYKDDDDK), Cell Signaling Technology, 14793S
Flag-Tag (DYKDDDDK), Proteintech, #20543-1-AP
GAPDH, Santa Cruz, SC-47724
HA-Tag, Cell Signaling Technology, #3724S
HA-Tag, Sigma, H3663
IGF2BP1 (IMP1), Cell Signaling Technology, #8482S
IGF2BP2 (IMP2), Cell Signaling Technology #14672S
IGF2BP3 (IMP3), Cell Signaling Technology #57145S
LC3B, Cell Signaling Technology, #3868S
METTL16, Cell Signaling Technology, #17676S
p62, Sigma, P0067
p62, Progen Biotechnik GmbH, GP62-C
TLR3, Cell Signaling Technology, #6961S
TLR3, Abcam, ab62566
TLR3, Abcam, ab13915,
YTHDC1, Abcam, ab122340
YTHDC2, Abcam ab176846
YTHDF1, Proteintech, #66745-1-Ig
YTHDF2, Abcam, ab220163
YTHDF2, Proteintech, #247441-1-AP
YTHDF2, Cell Signaling Technology, #80014S
YTHDF3, Cell Signaling Technology, #24206S
PARP1, Santa Cruz, sc-8007
Rab5, Abcam, ab18211
Rab7, Abcam, ab50533
YTHDF2 pS39 Ab, Generated by ABclonal
CNOT1, Proteintech, 66507-1-lg

ICC/IHC/IF
Alexa Fluor® 488 Donkey Anti-Guinea Pig IgG (H+L), Jackson ImmunoResearch, 706-545-148
Alexa Fluor® 488 Donkey Anti-Mouse IgG (H+L), Jackson ImmunoResearch, 715-545-150
Alexa Fluor® 488 Streptavidin, Jackson ImmunoResearch, 016-540-084
Alexa Fluor® 594 Donkey Anti-Rabbit IgG (H+L), Jackson ImmunoResearch, 711-585-152
Alexa Fluor® 488 IgG Fraction Monoclonal Mouse Anti-Digoxin, Jackson ImmunoResearch, 200-542-156
Pan-cytokeratin, Origene, BP5069
RAB7, Abcam, Ab50533
RAB7, Abcam, Ab137029
TLR3, Abcam, ab62566
TLR3, Abcam, ab13915
YTHDF2, Proteintech, #247441-1-AP
YTHDF2, Abcam, ab220163
dsRNA, SCIONS, 10010200
Cytokeratin 14, Abcam, Ab7800
Cytokeratin 10, Abcam, Ab76318
COX-2, Santa Cruz, sc-376861

Flow Cytometric Analysis
FITC anti-mouse CD103 Antibody, 2 E7, Biolegend, 121420
FITC anti-mouse CD3 Antibody, 17A2, Biolegend, 100203
FOXP3 Monoclonal Antibody, PE, NRRF-30, Thermo fisher, 12-4771-82

PerCP/Cy5.5 anti-mouse/human CD45R/B220 Antibody, RA3-6B2, Biolegend, 103236
PerCP/Cyanine5.5 anti-mouse/human CD11b Antibody, 101228, Biolegend, 101228
PE/Cy7 anti-mouse CD279 (PD-1) Antibody, 29F.1A12, Biolegend, 109110
PE/Cyanine7 anti-mouse/human CD44 Antibody, IM7, Biolegend, 103030
PE/Cyanine7 anti-mouse Ly-6C Antibody, HK1.4, Biolegend, 128018
APC anti-mouse CD11C Antibody, N418, Biolegend, 117310
APC anti-mouse CD62L Antibody, MEL-14, Biolegend, 104412
Brilliant Violet 421™ anti-mouse I-A/I-E Antibody, M5/114.15.2, Biolegend, 107632
Brilliant Violet 421™ anti-mouse NK-1.1 Antibody, PK136, Biolegend, 100451
Brilliant Violet 510™ anti-mouse Ly-6G Antibody, 1A8, Biolegend, 127633
Brilliant Violet 605™ anti-mouse CD4 Antibody, GK1.5, Biolegend, 108741
Brilliant Violet 650™ anti-mouse CD45 Antibody, 30-F11, Biolegend, 103151
Brilliant Violet 711™ anti-mouse CD8a Antibody, 53-6.7, Biolegend, 100759
APC Rat IgG2a, κ Isotype Ctrl Antibody RTK2758, Biolegend, 400512
PE/Dazzle™ 594 Armenian Hamster IgG Isotype Ctrl Antibody, HTK888, Biolegend, 400951
PerCP/Cy5.5 Syrian Hamster IgG Isotype Ctrl Antibody, SHG-1, Biolegend, 402027
FITC Armenian Hamster IgG Isotype Ctrl Antibody, HTK888, Biolegend, 400906
PE Mouse IgG1, κ Isotype Ctrl (ICFC) Antibody MOPC-21, Biolegend, 400140
PE/Cyanine7 Rat IgG2b, κ Isotype Ctrl Antibody RTK4530, Biolegend, 400618

Validation

The commercial antibodies were validated based on the information of the manufacturers' instructions and additionally the antibodies were validated by the use of negative control and/or positive control (such as knockdown or overexpression) for YTHDF2 and TLR3 antibodies.
The concentration recommended from antibody's date sheet was used for western blot, dot blot and immunostaining.

Eukaryotic cell lines

Policy information about cell lines and Sex and Gender in Research

Cell line source(s)

Human HaCaT keratinocytes were kindly provided by Prof. N. Fusenig [German Cancer Research Center (DKFZ), Heidelberg, Germany];
A431, HEK293T (human embryonic kidney), CHL-1, and HeLa cells are from ATCC;
Normal Human Epidermal Keratinocyte (NHEK) cells were purchased from Lonza;
MEF(mouse embryonic fibroblast), WT and ATG5 KO MEF cells were provided by Dr. Noboru Mizushima;
WT and ATG7 KO and p62 KO MEF cells were provided by Dr. Masaaki Komatsu.

Authentication

All cell lines were authenticated according to the ATCC cell line authentication test recommendations, including morphology check by microscope, growth curve analysis, and mycoplasma test.

Mycoplasma contamination

All cell lines were tested to be mycoplasma negative. All lines were routinely tested for mycoplasma contamination.

Commonly misidentified lines
(See ICLAC register)

No commonly misidentified cell lines listed by ICLAC were used.

Animals and other research organisms

Policy information about studies involving animals; ARRIVE guidelines recommended for reporting animal research, and Sex and Gender in Research

Laboratory animals

Athymic Nude mice (6-8 weeks of age) were obtained from Envigo.
NSG mice (severely combined immunodeficient (NOD/SCID) interleukin-2 receptor (IL-2R) gamma chain null) were obtained from Jackson Laboratory.
Mice with wild type (WT; YTHDF2 flox/lox, kindly provided by Dr. Chuan He) and conditional skin-specific YTHDF2 deletion (DF2 cKO, K14Cre;YTHDF2 flox/flox) in the SKH-1 background were generated used for UVB irradiation experiments.
WT (Ythdf2 flox/flox) and DF2 cHet (K14Cre; Ythdf2flox/+) primary keratinocytes were obtained from neonates mice and used for UVB irradiation experiments.
The Atg7fl/fl and p62fl/fl mice were provided by Dr. Masaaki Komatsu.
The Atg5fl/fl mice were provided by Dr. Noboru Mizushima.

Wild animals

No wild animal was used in this research.

Reporting on sex

Tumorigenesis analysis in nude mice was performed in female mice based on our previous experience (n=3). UV tumorigenesis experiment was performed in both male and female mice and similar effect of YTHDF2 deletion was detected. n=9 for each group for female mice; n=8 (WT) and n=9 (DF2 cKO) for male mice.

Field-collected samples

No field-collected samples were used.

Ethics oversight

All animal procedures used were approved by the University of Chicago institutional animal care and use committee.

Note that full information on the approval of the study protocol must also be provided in the manuscript.

Flow Cytometry

Plots

Confirm that:

☒ The axis labels state the marker and fluorochrome used (e.g. CD4-FITC).

☒ The axis scales are clearly visible. Include numbers along axes only for bottom left plot of group (a 'group' is an analysis of identical markers).

☒ All plots are contour plots with outliers or pseudocolor plots.

☒ A numerical value for number of cells or percentage (with statistics) is provided.

Methodology

Sample preparation	Single cell suspensions were prepared from spleen and skin tissues. Live/dead labeling was performed before the cell surface staining, using a Zombie NIR™ Fixable Viability Kit (Biolegend; catalog number 423106) diluted 1:1,000 in PBS for 10min at room temperature in dark. Cells were labeled with specific fluorescence-conjugated antibodies to CD45, CD3, CD4, CD8, MHCII, CD11b, CD103, Ly6C, and/or Ly6G for 20 min on ice. Intracellular cytokine staining was performed using an eBioscience Intracellular Fixation & Permeabilization Buffer Set (catalog number 88-8824-00) according to the manufacturer's instructions. Antibodies against FOXP3 (clone; NRRF-30) were used for regulatory T cell staining.
Instrument	Fortessa 4-15 (BD Bioscience) and Attune NxT (Thermo Fisher Scientific)
Software	Flowjo V10.6.1
Cell population abundance	N/A
Gating strategy	Live cells (Zombie NIR negative) were gated using Zombie-violet (Catalog:423105) staining. FSC-A and FSC-A to exclude doublets. Lymphocytes were gated on SSC-A and FSC-A. CD4+ and CD8+ TILs gated on CD45+CD3+ cells. Detailed gating strategies are shown in supplementary Figure S18.

☒ Tick this box to confirm that a figure exemplifying the gating strategy is provided in the Supplementary Information.
